# Cellulose-Based Carbon Molecular Sieve Membranes for Gas Separation: A Review

**DOI:** 10.3390/molecules25153532

**Published:** 2020-08-01

**Authors:** Tiago Araújo, Gabriel Bernardo, Adélio Mendes

**Affiliations:** LEPABE-Laboratory for Process Engineering, Environment, Biotechnology and Energy, Faculty of Engineering, University of Porto, Rua Dr. Roberto Frias, 4200-465 Porto, Portugal; tjtbma@fe.up.pt (T.A.); gbernardo@fe.up.pt (G.B.)

**Keywords:** membranes, cellulose, carbon molecular sieve membranes, Robeson Index

## Abstract

In the field of gas separation and purification, membrane technologies compete with conventional purification processes on the basis of technical, economic and environmental factors. In this context, there is a growing interest in the development of carbon molecular sieve membranes (CMSM) due to their higher permeability and selectivity and higher stability in corrosive and high temperature environments. However, the industrial use of CMSM has been thus far hindered mostly by their relative instability in the presence of water vapor, present in a large number of process streams, as well as by the high cost of polymeric precursors such as polyimide. In this context, cellulosic precursors appear as very promising alternatives, especially targeting the production of CMSM for the separation of O_2_/N_2_ and CO_2_/CH_4_. For these two gas separations, cellulose-based CMSM have demonstrated performances well above the Robeson upper bound and above the performance of CMSM based on other polymeric precursors. Furthermore, cellulose is an inexpensive bio-renewable feed-stock highly abundant on Earth. This article reviews the major fabrication aspects of cellulose-based CMSM. Additionally, this article suggests a new tool to characterize the membrane performance, the Robeson Index. The Robeson Index, θ, is the ratio between the actual selectivity at the Robeson plot and the corresponding selectivity—for the same permeability—of the Robeson upper bound; the Robeson Index measures how far the actual point is from the upper bound.

## 1. Introduction

Nowadays, most industrial gas separations rely on energy demanding and expensive processes such as cryogenic distillation and pressure swing adsorption. As stated by Sholl et al. [1] in the journal *Nature*, more energy-efficient separation processes methods could save 100 × 10^6^ tons of carbon dioxide emissions per year if applied to the US alone. In this context, membrane separation appears as a promising industrial process set to reduce the energy intensity of the separation process.

Although the first scientific study on membrane-based separations dates back to the 18th century, significant developments in membrane technology were made only after the World War II to make viable for the commercial market, and in the 1960s, high-flux anisotropic membrane modules were applied for reverse osmosis applications [2,3]. In 1980, the company Permea (now Air Products and Chemicals) adapted this technology for gas separation for the first time with polysulfone hollow fiber membranes.

Membrane technologies for gas separations have been growing ever since. They present several advantages, such as continuous separation, low energy cost, easy scale-up and easy coupling with other separation processes in an industrial environment. Studies predict that the technology should grow from 3.8 billion dollars in 2018 to 5.1 billion dollars in 2023 [4]. Due to the great demand for cost-effective solutions to carbon dioxide removal from natural gas and biogas, varoious membrane separation processes have emerged, providing growth in the market size: USD 846 million in 2019, projected to reach 1131.6 million USD by 2024 [5].

The potential gas separation markets for the use of membrane separation technologies include CO_2_ removal, H_2_ recovery, ethane/ethylene and propane/propylene separation, nitrogen generation and oxygen and nitrogen enrichments. Other separations, such as sour gas treatment, Xe recovery, He separation or sulfur removal, have been investigated and promising results have been achieved with membrane technology [5].

This review is focused on a very specific type of membranes for gas separation and of great interest and market potential: carbon molecular sieve membranes based on cellulosic precursors [6,7,8,9,10]. The review starts with a very brief introduction to membrane technology; then, carbon molecular sieve membranes (CMSM) are presented and the cellulose as a promising lignocellulosic material is used as a CMSM precursor. Finally, a more detailed review on the major developments on cellulose-based CMSM is presented.

### 1.1. Membranes

**Figure 1:**
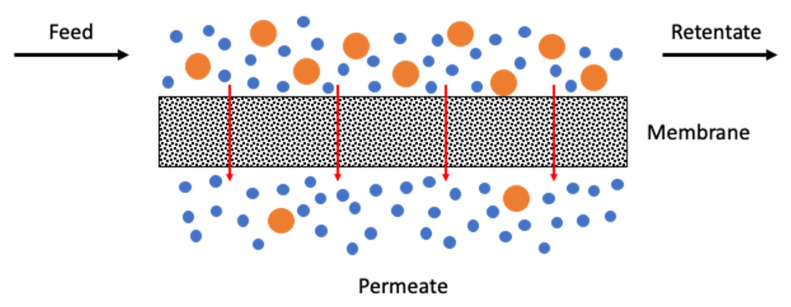
Scheme of a membrane separation.

A membrane can be defined as a selective barrier between two streams or an interface between two phases [11], see Figure 1. The membrane is the nucleus of a membrane separation process. This separation process consists of dividing the feed stream into two streams: the retentate stream and the permeate stream. The final product to be separated can be obtained in the permeate stream or in the retentate (or concentrate) stream. The separation occurs because the membrane is selective towards one or more components of the feed stream, allowing them to pass through. In gas separation applications, the gradient in chemical potential between both sides of the membrane is the force that drives the different species to permeate a membrane. This gradient in chemical potential can be translated into partial pressure difference.

The membrane performance is characterized by two parameters: the membrane productivity, named permeability, and the membrane selectivity, which represents the membrane separation efficiency. Currently, there are several gas separation membrane technologies at different stages of development and industrial implementation and these include the polymeric membranes, the palladium-based membranes and carbon molecular sieve membranes (CMSM), among others [11,12].

**Figure 2:**
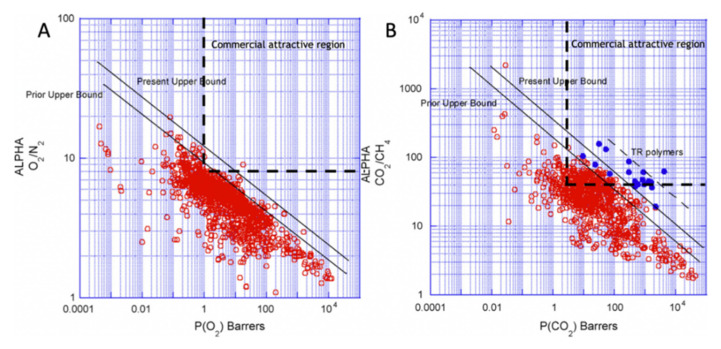
Robeson upper bound plots for O_2_/N_2_ separation (**A**) and for CO_2_/CH_4_ separation (**B**) with their industrial application attractive region, where the carbon molecular sieve membranes (CMSMs) are included (adapted with permission from [15,17]).

The polymeric membranes, due to their easy to process, low production cost and high mechanical stability, are the most-used type of membranes [13]. These membranes operate normally through a sorption-diffusion mechanism, but they suffer from some important limitations. Robeson published three studies, with different target gas separation results, demonstrating that in polymeric membranes, permeability and selectivity are inversely related, existing an intrinsic experimental upper bound limit, known as Robeson upper bound, for the separation performance of these type of membranes [14,15,16]. As shown in Figure 2, no experimental data points can be found above the Robeson upper bound limit. This balance between permeability and selectivity remains one of the greatest challenges in the production and investigation of membranes. Furthermore, due to the thermal transition and the decomposition of the polymer, the polymeric membranes are inappropriate for high temperature applications and they are susceptible to corrosion and other chemical aging.

Contrary to polymeric membranes, the performance of the carbon molecular sieve membranes (CMSMs) is not limited by the Robeson upper bound and can present more favorable balances between permeability and selectivity well above that upper bound. Furthermore, CMSMs also present high chemical resistance in chemically aggressive environments and resistance to high temperatures, which, combined with the vast amount of low cost and available precursors, such as cellulose, makes the production of these membranes economically attractive. For air separation, the maximum temperature that CMSM can withstand is ~200 °C. In the case of CO_2_ separation, thermal stability is maintained up to ~400 °C. H_2_ and He separations can be carried out at temperatures above 500 °C [18]. However, despite several attempts, CMSM are still not commercially available. We briefly describe CMSM in the next section.

### 1.2. Carbon Molecular Sieve Membranes

Carbon molecular sieve membranes (CMSM) are prepared by the thermal decomposition, at high temperatures, of polymeric precursor materials under controlled conditions [19]. The concept of CMSM for gas separation appeared in the 1970s and the first crack-free CMSMs produced by the thermal decomposition of several polymeric precursors were reported by Koresh and Soffer in the 1980s [20,21].

**Figure 3:**
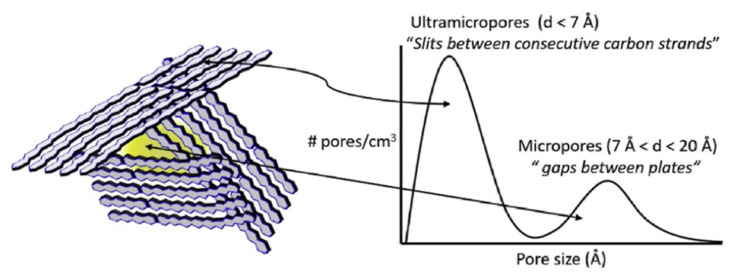
Turbostratic CMSM structure with idealized pore size distribution (adapted with permission from [26]).

After carbonization, the CMSM presents a structure with disordered sp2 hybridized carbon sheets packed imperfectly—a graphitic structure [22]. The graphite pack results in an amorphous disordered structure where pores are formed in the imperfection between the crystalline regions [23]. The CMSM structure can be described as a turbostratic ribbon-like structure that is also isotropic [23,24]. The pore structure can be described as “slit-like” with a bimodal pore size distribution (Figure 3) with micropores connecting ultramicropores [25]. Micropores (0.7-2.0 nm) provide sorption sites responsible for the surface diffusion, while ultramicropores (<0.7 nm), also called constrictions, are enable for molecular sieving, making CMSM both highly permeable and highly selective—a distinct characteristic of these materials [25]. This type of inorganic membranes presents high permeability due to the micropores that offer the sorption sites and long jump lengths. In these sorption sites, the gas transport occurs due to the molecule jumps from one sorption site to the next, providing a concentration gradient; a high selectivity is ensured by the ultramicropores, which restrict the diffusion of the larger molecules.

The predominant mass transfer mechanism in CMSMs is the molecular sieving mechanism (as the membrane name suggests). As the ultramicropores are very narrow (3–5 Å), the smaller diameter gas molecules permeate through the ultramicropores and the larger molecules are retained, giving it higher membrane selectivity when compared with other mechanisms [27].

**Figure 4:**
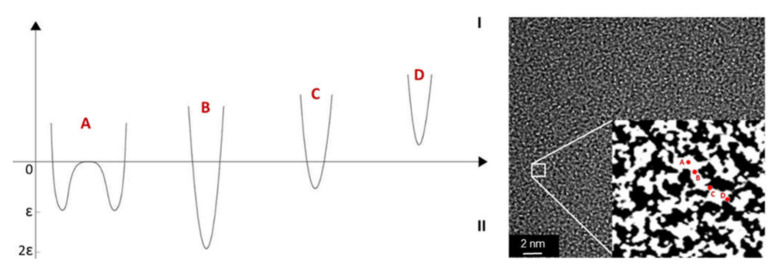
(I) Graphic representation of the gas molecules interaction within a pore or constriction. (**A**) There is no overlap of the potential associated with the opposite walls because the pore is too large; (**B**) As the opening of the pore increases, maximum absorption energy is reached, and the potential reaches a minimum; (**C**) The pore becomes a constriction and the sorption energy is smaller; (**D**) The potential becomes positive and the pore is impervious to the species; (II) real pore configuration (adapted with permission from [28]).

In the CMSM pores, the gas molecules are confined to the space of the pore diameter and subjected to a potential energy field [28]. Figure 4 shows the relative potential of molecule within a pore. In case A, the pore is large enough, and thus, the minimum potential occurs autonomously for each wall. The permeating species stays in the adsorbed phase; as the pore narrows, the potential crosses a minimum, increasing afterwards until becoming impenetrable to the species (case D). However, if repulsive forces in the potential field dominate, the sorption energy is smaller, and the pore becomes a constriction that the diffusing molecules must overcome (case C). Different gas molecules can show different sorption behaviors when interacting with the same pore [28,29].

**Figure 5:**
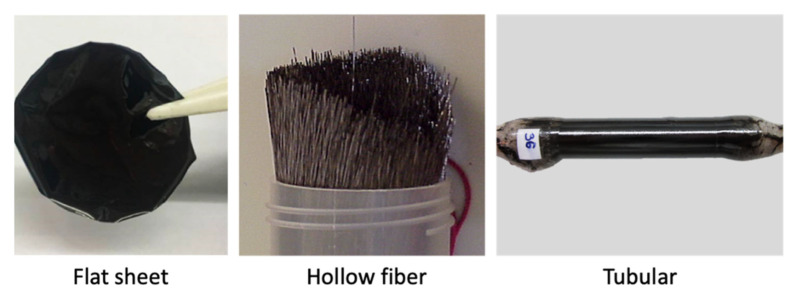
Different geometries of CMSM (adapted with permission from [30]).

A CMSM can be either self-supported or supported in a porous and mechanically stable material [23]. The supported membranes can be flat or tubular while the unsupported membranes can be flat, capillary or hollow fibers (Figure 5).

**Figure 6:**
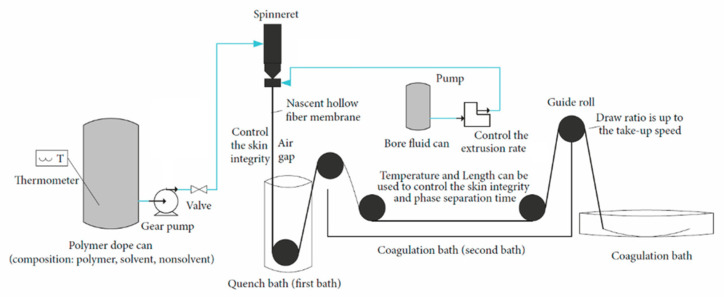
Scheme of the hollow fiber dry-wet spinning machine (reproduced with permission from [31]).

Hollow fiber CMSMs are produced by the carbonization of hollow fiber polymer precursor membranes produced by dry-wet spinning. The dry-wet spinning method (Figure 6) includes two fluids: the dope solution that contains the polymer precursor and the bore fluid, i.e., the fluid responsible for creating the inner wall of the hollow fiber. This processing method involves several adjustable parameters that can influence the structure of the polymeric fiber and results in hollow fiber CMSM with different structures and separation performances.

Aiming to increase the CMSM pore volume, pore size and surface area, composite materials have been developed [32,33]. A composite Carbon Molecular Sieve Membrane (c-CMSM) is formed by the incorporation of inorganic compounds in the carbon structure [32]. The inorganic compounds that are used in the polymeric precursor must have an affinity with the matrix. In 2010, Foley et al. patented a method of fabrication of a thin nanocomposite CMSM [33], where the incorporation of inorganic nanoparticles allowed to achieve higher permeances. Some metals, such as Ag, have been shown to be very effective at increasing the separation performance of CMSM [34]. Zeolites can also be added to the polymeric precursors of CMSM to increase their separation performance [35,36].

Here, we hypothesize on the important role that can be played by small molecules (additives, plasticizers) on the formation of homogeneous empty spaces (porosities) in the amorphous carbonized matrix of CMSM. In the authors’ opinion, this important role has thus far been overlooked; hereafter, in this review, these small molecules will be designated as “molecular spacers.” Broadly speaking, molecular spacers can be either small molecules with high boiling temperatures or polymers/oligomers with relatively small thermal decomposition temperatures, which are homogeneously blended with the polymer precursors that, during the carbonization process, are released from the precursor matrix at an early stage leaving a network of pores with a narrow pore size distribution. The release of molecular spacers during carbonization may occur by either of the following two mechanisms: (i) simple evaporation, only possible with small molecules, in which case the pores formed have dimensions similar than the original molecular spacers or aggregates of molecular spacers; (ii) degradation followed by partial volatilization of the corresponding molecular fragments, in which case the carbonaceous residual is trapped inside the pores causing smaller pore sizes. In practice, molecular spacers should evaporate or decompose at temperatures below the carbonization end temperature of the polymer precursors.

The CMSM pore structure can be tailored for the desired gas separation application. Their production usually includes the following steps: (i) selection of the polymeric precursor; (ii) pre-treatments; (iii) carbonization; (iv) post-treatments [37]. Small changes in these steps can largely influence the final CMSM pore structure and separation performance. The influence of these steps, on the performance of cellulose-based CMSM, is thoroughly described and discussed in Section 3 of this review.

### 1.3. Robeson index—A New Figure of Merit

The authors of this review propose here a new figure of merit, the Robeson Index, which will be used in this review to characterize the separation performance of CMSM.

The Robeson correlation for the upper bound limit is described by the following relationship:

(1) $$L_{i}=k\alpha_{i,j}^{n}$$

where $L_{i}$ is the permeability of the fastest gas, $\alpha_{i,j}$ is the ideal selectivity, k is the “front factor” and n is the slope of the log-log relationship [14].

Freeman predicted theoretically the empirical upper bound relationship in agreement with the experimental data previously compiled [38]. The slope of the upper bound limit, −1n, was shown to be related, according to activation energy theory, to the difference between the gas molecular diameters by:

(2) $$\frac{-1}{n}={\left(\frac{d_{j}}{d_{i}}\right)}^{2}-1=\left[\frac{d_{j}+d_{i}}{d_{i}{}^{2}}\right]\left(d_{j}-d_{i}\right)$$

The plot of the relationship −1n~(dj−di) is linear and passes through the (0,0) for the x-y plot, thus providing further verification of this analysis [15,16,38]. Freeman [38] also predicted the *k* value using the following equation:

(3) $$k^{-1/n}=\frac{S_{i}}{S_{j}}S_{i}^{-1/n}\exp\left\{\frac{1}{n}\left[b-f\left(\frac{1-a}{RT}\right)\right]\right\}$$

where $S_{i}$ and $S_{j}$ are the sorption constants for gases i and j, a = 0.64, b = 9.2 for rubbery polymers and b = 11.5 for glassy polymers and f is a polymer dependent constant set to be 12,600 cal·mol^−1^ to provide the best fit to the upper bound data [38]. The experimental and predicted values of the Robeson upper bound correlation are presented in the Table 1. All the presented values are related to the revised upper bound with the exceptions of H_2_/O_2_ and He/O_2_ systems.

To compare the experimental values of the permeability vs. the selectivity (separation performance of the membranes) with the upper limit of Robeson, the authors suggest for the first time, a figure of merit, θ, named Robeson Index. The Robeson Index is the ratio between the actual selectivity value and the one corresponding to the Robeson upper bound ($\alpha_{i,j|RUB}$) both for a given permeability:

(4) $$\theta =\frac{\alpha_{i,j}}{\alpha_{i,j|\mathrm{RUB}}}=\frac{\alpha_{i,j}}{k^{-1/n}\;L_{i}^{1/n}}=\frac{1}{\beta }\frac{\alpha_{i,j}}{L_{i}^{m}}$$

where $L_{i}$ is the permeability of the fastest gas and $\alpha_{i,j}$ is the ideal selectivity, both corresponding to the membrane, m is the slope of the Robeson Upper Bound for the actual gas pair and it is equal to the slope of the log-log of the relationship and β is the y-intercept of the Robeson upper bound and it is equal to the Freeman in Equation (3).

The Robeson index teaches how many times the performance of the actual membrane is from Robeson’s upper bound limit; membranes with performances above and below the Robeson upper bound have θ values of >1 and <1, respectively. The Robeson β values for several gas mixtures is given in Table 2.

In this review, the performance of CMSM will be also characterized by the Robeson Index, thus demonstrating the usefulness of this figure of merit. The separation performance of all the types of membranes can be characterized by the Robeson Index.

## 2. Cellulosic Precursors for CMSM

Carbon molecular sieve membranes (CMSM) are prepared from the thermal decomposition of polymeric precursors. The selection of polymer precursor plays a crucial role on the CMSM production [27]. This selection determines the final structure of the CMSM as different polymer precursors carbonized in the same conditions can result in carbon membranes with different properties [39].

Numerous research groups have been investigating different thermosetting polymers as precursors for CMSM [40]. This polymer family does not liquefy or soften up to the carbonization temperature, a required specification to produce CMSM [37]. The choice of the polymeric precursor depends on the required gas mixture to be purified. Among the most promising polymer precursors are the polyimides [41,42,43], due to their high thermal stability, commercially availability and the ease of processing their membranes, but their price is still very high. Other polymer precursors such as polyacrylonitrile (PAN) [44,45], polyfurfuryl alcohol (PFA) [46,47], phenolic resins [48,49,50,51], polyphenylene oxide (PPO) [52,53,54], polyetherimide (PEI) [55,56] and cellulose have been used to produce CMSM for gas separation.

**Figure 7:**
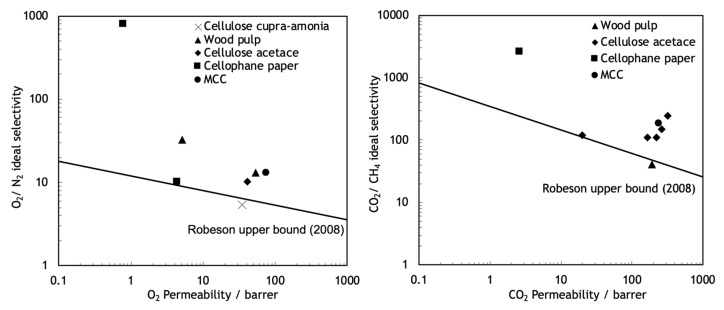
Robeson plot for O_2_/N_2_ and CO_2_/CH_4_ separation with the best cellulose-based CMSM results.

Cellulosic precursors display several advantages over other polymeric precursors including a higher availability, a lower cost and an environmentally-friendly processing. Furthermore, cellulose-based CMSMs, compared with CMSMs derived from other precursors, have higher permeability and selectivity to several permanent gases such as natural gas separations [30,57,58,59], as can be seen in Figure 7.

Cellulose is the most abundant organic compound on Earth, being the main structural component of plants. For example, cotton is almost pure cellulose and wood is cellulose combined with lignin. Cellulose is regularly regenerated by nature in relatively short time periods; therefore, it is an inexpensive and nearly inexhaustible raw material. Chemically, cellulose is a polysaccharide of D-glucopyranose residues connected by β-1,4-glycosidic linkages.

**Figure 8:**
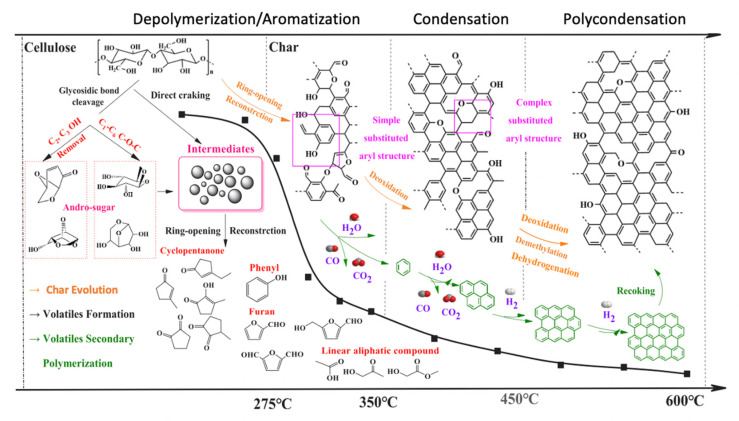
Schematic diagram of cellulose pyrolysis mechanism (readapted with permission from [60]).

Cellulose is a thermosetting polymer and then does not melt during the carbonization processes. Thus, high temperature heating of cellulose, even in an inert atmosphere, results in a complex series of chemical reactions. The pyrolysis mechanism of cellulose has been previously studied by different authors [60,61,62,63]. Among these studies, one deserves special attention, namely a very recent work by Yang et al. [60], who studied the carbonization of cellulose in the temperature range 200–600 °C using two-dimensional perturbation correlation infrared spectroscopy (2D-PCIS). The cellulose carbonization process is represented in Figure 8. In the lower temperature range (≤275 °C), some cracking of C-OH and C-H structures occurs around the pyran ring, but the molecular and crystal structure of cellulose suffers only minor changes. With the temperature range increasing, 275−350 °C, some glycosidic bonds are broken to form dehydrated sugars and pyranone products and the crystal structure of cellulose is completely decomposed. At 350 °C, the char consists mainly of aromatic and alicyclic compounds rich in C=O groups. In the next temperature range (350−450 °C), glycosidic bonds are rapidly broken, decarbonylation reactions of char take place with largely increased amounts of CO and CO_2_ released as gas products. At the same time, substitution reactions between aromatic rings in the char create an amorphous three-dimensional (3D) network structure containing a large number of low-order fused rings (two−five rings). Finally, in the range 450−600 °C, a further removal of oxygen occurs, containing groups and alkyl structures in the char, creating gas products such as CO and CH_4_. Dehydrogenation and condensation reactions between benzene rings and dense small-molecule rings form higher-order fused rings with a release of a large amount of H_2_.

Cellulose is a strongly hydrogen-bonded system with a very limited solubility in usual solvents. Research in cellulose-based regenerated fibers has been largely driven by the textile industry. Regenerated cellulosic fibers utilize natural cellulose as the raw material and are processed by either direct dissolution or by derivatizing cellulose [64]. In non-derivatizing process, cellulose is directly dissolved without modification; cuprammonium, LiCl/DMAc and Lyocell (with NMMO solvent or ionic liquids) are examples of this process. By contrast, in the derivatizing process, cellulose is modified before dissolution; viscose and cellulose acetate are examples of this regeneration process.

The ionic liquids represent a particularly promising alternative to existing cellulose-dissolving solvents. Ionic liquids are a group of salts with low melting point, low vapor pressure, excellent dissolution ability, high thermal and chemical stability and non-inflammable [65,66,67]. Due to their low tendency to crystalize they are suitable solvents to dissolve cellulose in the production of tailor-made cellulosic precursors.

## 3. Cellulose-Based Carbon Molecular Sieve Membranes

### 3.1. Selection of Cellulosic Precursor

The first CMSM reported in the literature was prepared from the carbonization of cuprammonium cellulose hollow fibers by Koresh and Soffer in 1983 [20]. Later, in 1987, the same authors patented these membranes for gas separation applications [68] and in 1995, they patented the method to produce the cellulose-based carbon hollow fiber membranes [69]. At the end of the 1990s, Israeli company Carbon Membranes Ltd. bought these patents and became the first company in world to produce and commercialize modules with carbon hollow fiber membranes for gas separation, produced from a cellulosic precursor. However, in 2001, the company went bankrupt and was forced to shut down. Mendes and his team [70,71] have evaluated the separation performance and the aging effect of their modules.

Lagorsse et al. [70] studied the sorption and transport properties of the CMSM produced by Carbon Membranes Lda. These membranes were made by a precursor of cellulose cuprammonium and a thin carbon film was applied by chemical vapor deposition (CVD) to improve the membrane selectivity. This treatment is discussed in detail in Section 3.4. The commercial CMSM tested, exhibited permeability to He, CO_2_ and O_2_ of up to 107, 183 and 35 barrer, respectively, and O_2_/N_2_ and CO_2_/N_2_ ideal selectivity of 5 and 30, respectively. The membrane permeance to different gases was found to increase with the test temperature and decrease with the feed pressure [7,72,73,74]. The permeance to CO_2_ decreases due to the strong concentration dependence of the CO_2_ diffusion coefficient [70]. Koros suggested that this decrease is due to the fact that interactions between adsorbate/adsorbent increase; due to the sorption effects and the non-idealities of the gas phase at high pressures [72]. The mass transport mechanism of CMSM can be described by the sorption diffusion mechanism and the CO_2_ adsorption isotherm follows Langmuir shape [72].

Wood pulp, also known as “kraft,” composed by a cellulose:hemicellulose mass ratio of 4:1 was used as a polymeric precursor, for the first time, by Lie et al. in 2005 [57], to produce selective carbon membranes. The wood pulp membranes were produced by the dissolution of the cellulose in trifluoroacetic acid (TFA), to a concentration of ~1 wt%. The TFA exposure time was found to affect the separation performance as discussed in Section 2. These unsupported flat sheet membranes displayed a selectivity to O_2_/N_2_ of 13, well above the 2008 Robeson Upper Bond [15], with a permeability of O_2_ of 54 barrer, presenting a Robeson Index of 2.2. The permeability of CO_2_ was 190 barrer with a CO_2_/CH_4_ selectivity of 41 and a θ of 0.85.

In 2010, Mendes et al. reported a promising precursor for CMSM, the cellophane paper [58]. The cellophane paper is made of regenerated cellulose obtained by the viscose process. A commercial cellophane paper was carbonized in a single carbonization step under N_2_ atmosphere and the produced flat sheet CMSM presented no cracks and defects and a good mechanical resistance. Maximum permeability was obtained with a carbonization end temperature of 550 °C: O_2_ permeability of 4.3 barrer with a O_2_/N_2_ selectivity of ~13 displaying a θ of 0.94. The permeability of these membranes is low compared to other cellulose-based CMSM due to a low micropore volume, i.e., a small number of pores.

**Figure 9:**
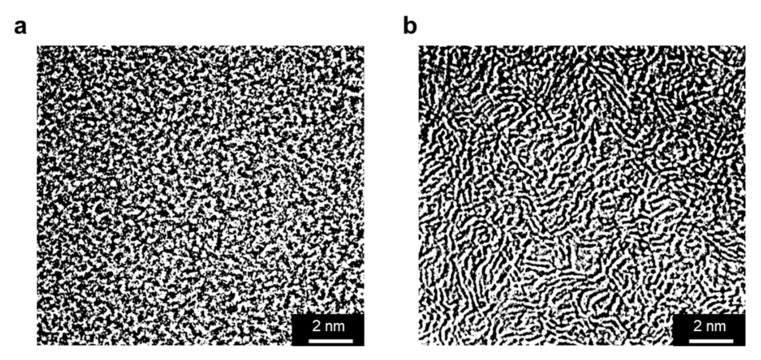
HRTEM images of: (**a**) CMSM 550 sample (gate sieving) and (**b**) CMS adsorbent (tubular sieving) (reproduced with permission from [75]).

Recently, in 2019, the same group reported a new work on CMSM based on cellophane paper precursor [75]. Rodrigues et al. [75] presented a CMSM with extremely high separation performance: the CMSM carbonized at 600 °C is situated far above Robeson’s upper bound, showing a O_2_/N_2_ selectivity greater than 800, a CO_2_/CH_4_ selectivity greater than 2600 and a H_2_/CH_4_ selectivity greater than 25,000, but with low permeability. These cellulose-based CMSM shows the highest Robeson Index values for the O_2_/N_2_ and CO_2/_CH_4_ separations, 63 and 100, respectively. X-ray photoelectron spectroscopy (XPS) and Inductively coupled plasma (ICP) analyses proved the presence of metallic and semi-metallic groups (ionic sodium and silica nanoparticles) homogeneously distributed on the membrane surface, providing polar sites that allow the water molecules to jump between them avoiding the formation of water clusters [75]. This confers a very high humidity stability to these membranes (absence of pore blockage) and a very high water vapor permeability. The permeability of C_3_H_6_ and C_3_H_8_ was also measured, but the ideal selectivity attained was very low compared to the literature values for this separation. The cellulose-based CMSM nanostructure was compared, as studied by High-resolution transmission electron microscopy (HR-TEM), with the nanostructure of a phenolic-resin based CMS adsorbent produced by the same authors and displaying, respectively, high and low ideal selectivity to C_3_H_6_/C_3_H_8_ and O_2_/N_2_ mixtures. Interestingly, the membrane sample with high O_2_/N_2_ selectivity presented a morphology with gate-like pores, which is characteristic of a gate sieving mechanism, see Figure 9a, whereas the CMS adsorbent presented interconnected pores in form of earthworm-like pores, characteristic of a tubular sieving mechanism, see Figure 9b.

In a series of works, May-Britt Hägg et al. produced hollow fiber CMSM by a dry-wet spinning method using as polymer precursor cellulose acetate (CA), from ACROS, with a molecular mass (MM) of 100,000 g·mol^−1^ and an average acetyl content of 39.8% [31,76,77,78,79,80,81]. This acetyl content was later removed by a deacetylation process.

The gas separation performance for carbon hollow fiber resulting from the precursor casting of CA/PVP/NMP (22.5%/5%/72.5%), where PVP is poly-vinylpyrrolidone and NMP is *N*-methyl-2-pyrrolidone, presents an O_2_/N_2_ selectivity of 10 with an O_2_ permeability of 41 barrer and a CO_2_ permeability of 220 with a CO_2_/CH_4_ ideal selectivity up to 110 [77] with a θ value of 1.6 and 2.1, respectively. A H_2_ permeability of 980 barrer with a H_2_/CH_4_ permselectivity of 490 [76] displays a Robeson Index of 24.3.

He [79] optimized the dry-wet spinning parameters such as air gap (distance from the spinneret to the coagulation bath), bore fluid composition, flow rate of the bore fluid and the quench bath temperature in the fabrication of defect-free cellulose acetate hollow fibers. The dope solution consisted of cellulose acetate (CA) precursor and the additive poly-vinylpyrrolidone (PVP) in *N*-methyl-2-pyrrolidone (NMP). The bore fluid consisted of either water or a water:NMP mixture (85%). The optimum conditions reached were a bore fluid with a water:NMP mixture, a spinneret-quench bath air gap of 25 mm, a bore fluid flow rate equal to 40% of the dope rate and a temperature of quench bath of 50 °C. These conditions allowed the production of defect-free CA based carbon hollow fiber precursors with a high PVP content, symmetrical cross-section and high cross-linking degree between the cellulose and the PVP.

The industrial application of the cellulose acetate-based carbon hollow fibers was evaluated by a DPCOI (Project-Preparation-Operation-Integration) platform. This tool was used to connect the fundamental researches and applications from the material conception into the industrial application. The results of HYSYS simulations confirmed the potential of the application of these hollow fiber CMSM for CO_2_ capture in an industrial plant [78].

A pilot-scale production of carbon hollow fiber membranes from regenerated cellulose acetate precursor and with an annual production capacity 700 m^2^ of carbon membrane was reported by Haider [80,81]. A dope solution of cellulose acetate (CA) mixed with PVP and NMP and bore fluid of NMP and water was used in the dry-wet spinning process. The bore fluid composition was changed (65, 70, 80 and 85% of NMP in H_2_O; 85, 90 and 95% of DMSO in H_2_O) to investigate the influence of the non-solvent in the membrane separation performance after carbonization. The permeability of CO_2_ reached a maximum value of 256 barrer for 65% of NMP and decreased with the NMP content in the bore solution; the corresponding CO_2_/CH_4_ ideal selectivity remained approximately constant with the NMP content (156 and θ = 3.7 for 65% NMP). The carbon membranes prepared from hollow fiber precursors spun using a bore fluid of DMSO:H_2_O, exhibited overall lower performances: the best were prepared using 95% DMSO and exhibited a permeability of CO_2_ of 19 Barrer and a CO_2_/CH_4_ ideal selectivity of 220 (θ = 1.9) [80].

Despite the great expectation of the cellophane-based paper carbon membranes [75], such as its very high selectivity, >800 for O_2_/N_2_, the permeability of O_2_ is low (1 barrer). To overcome this limitation, Rodrigues et al. developed a high-performance cellulose-based CMSM prepared from an optimized ionic liquid-regenerated cellulose precursor [30]. In this work, 9.2 wt% of wood pulp was dissolved in DMSO and 1-ethyl-3-methyl imidazolium acetate (EMIMAc) to obtain a homogeneous solution. The precursor film was deposited, from this solution, by spin-coating. The spin-coated films were coagulated and washed in water to obtain a transparent regenerated cellulose film. The water bath was used to remove the excess of ionic liquid. Once washed, the cellulose films were dipped for 1 min in a softener bath containing 5 wt% of propylene glycol to obtain a plasticized film with a non-curling effect after drying. The ionic liquid-regenerated cellulose-based CMSM was made in one-step carbonization under N_2_ atmosphere producing a uniform membrane with a thickness of circa 20 µm and a defect-free smooth surface. This novel precursor displayed a very good permeation performance, well above Robeson upper bound for polymeric membranes. The membrane produced at 550 °C has displayed a permeability of O_2_ of 5.2 barrer and an O_2_/N_2_ ideal selectivity of 32.3, showing a better permeability/selectivity balance when compared with other cellulose-based CMSM with a θ of 3.6 [30]. The hydrophilic characteristic [58,75,82] that prevents the pore blockage when permeating humidified gas streams was preserved in this CMSM.

Lei et al. [83] used a microcrystalline cellulose (MCC) precursor to spun hollow fibers through a dry-wet spinning method, with a dope solution of 10 wt% of MCC with a co-solvent of 75 wt% EmimAc/25 wt% DMSO and a bore solution containing 20 wt% water/60 wt% EmimAc/20 wt% DMSO. From thermogravimetric analysis they concluded that produced MCC hollow fibers presented a pyrolysis behavior similar to deacetylated cellulose acetate.

Later, the same authors produced carbon hollow fiber membranes directly spun from a dope solution of 12 wt% of MCC dissolved in 75 wt% EmimAc/25 wt% DMSO [73]. After IL removal, the produced fibers were placed in a 10% glycerol aqueous solution with the same purpose as Rodrigues et al. when they put their films in a softener bath of propylene glycol. The membranes were carbonized under CO_2_ atmosphere. The authors investigated the influence of the spinning parameters on the produced carbon membranes. The permeability of CO_2_ increased with the air gap and the dope flow but decreased with the water content in this solution. A membrane permeability of CO_2_ of 239 barrer and a CO_2_/CH_4_ selectivity of 186 with a θ of 4.2 were obtained. The CO_2_/CH_4_ selectivity increased with the dope flow but decreased with the bore flow and the take up speed. Oxygen separation was also evaluated, and the membranes displayed a permeability of O_2_ of 74 with a O_2_/N_2_ selectivity of 13. The O_2_ membrane permeability is 15 times higher than the value obtained with the flat sheet membrane produced by Rodrigues et al. The Robeson Index for this CMSM is lower (2.3) compared with the Rodrigues et al. CMSMs for this separation (3.6).

Cellulose in its crystalline form, either nanocrystalline (NCC) or microcrystalline (MCC), has also been used as a thermally labile additive in polyimide based CMSM [84,85,86,87]. In 2017, Sazali et al. [84] prepared a supported CMSM from a polyimide precursor blended with nanocrystalline cellulose (NCC) dissolved in NMP. The NCC was synthesized from recycled newspaper and was used as a pore-forming agent for the CMSM. The results showed that the addition of NCC tends to increase the porosity and decrease the pore size distribution. Different NCC compositions were tested in polyimide, and the 7 wt% of NCC displayed higher permeance to H_2_ and higher selectivity. MCC was also added to this supported CMSM, but the carbon membranes prepared with NCC displayed higher permeance and selectivity [85]. The same author studied the influence in intermediate layers in the tubular carbon membrane for the gas separation performance [87]. The three different supports studied were: alumina powder, a carbon pencil and a carbon molecular sieve. A high separation performance was reached with alumina powder due to its smoother surface compared to the other layers.

The gas separation results of different cellulosic precursor based CMSM are summarized in Table 3.

### 3.2. Effect of the Pre-Treatments

The polymer precursors are often subjected to chemical and physical pre-treatments, i.e., treatments before the carbonization procedure, to stabilize the structure of the polymers during the carbonization and to enhance the mechanical properties of the produced CMSM [37]. The specific pre-treatment modifies the separation performance of the produced CMSM [88].

Physical pre-treatments are often applied in the case of hollow fibers and typically consist of stretching of the fibers immediately after the spinning process [29]. This pre-treatment removes surface imperfections and confers a better dimensional stability to the hollow fibers, improving the molecular orientation and attenuating diameter variations [23].

The chemical treatments are the most frequently employed on the manufacture of CMSM. Oxidation and/or thermostabilization pre-treatments are used to promote the crosslinking of some polymer precursors, thus, the polymer structure becomes stiffer, and in turn less prone to relaxation during the carbonization [23]. Exposure of the CMSM to atmospheric oxygen is also known to modify its separation performance. Other chemical treatments used include the immersion of the precursors in some chemical solutions, to improve porosity [69] and to obtain carbon membranes with higher carbon contents [37]. Some chemicals such as phosphoric acid, hydrochloric acid or ammonium chloride [69] act as catalysts on the pyrolysis reaction, allowing the carbonization to be performed at lower temperatures and faster heating rates, increasing the carbon yield [23].

In carbon membranes made from cellulose, these pre-treatments are also applied systematically. Lie et al. have hydrolyzed wood pulp in trifluoracetic acid (TFA) to produce cellulose-based CMSM [57,89]. Various hydrolysis pre-treatment times, defined as the time from cellulose dissolution in TFA until film drying in a vacuum oven, were tested (6, 14 and 74 days), and their influence on the membrane permeability/selectivity was assessed. An increase in the exposure time to TFA was found to increase the carbon yield during carbonization, i.e., decrease the mass loss, leading to a general decrease in the permeability. However, this was accompanied by large increase of the selectivity for selected gas pairs and a general increase in the separation performance. For example, CMSM with hydrolysis pre-treatment time of 74 days displayed performance for CO_2_/CH_4_ separation, well above Robeson’s upper bound with a Robeson Index of 2.2. By contrast, TFA exposure for just 6 days resulted in non-selective CMSM with CO_2_/CH_4_ separation performance well below Robeson’s upper bound (θ = 0.1). These results also demonstrated that furans released during carbonization play an important role as intermediates in the formation of microporosity [89].

The cellulose acetate precursor used by some authors has an average acetyl content of ~40% [31,76,77,78,79,80,81,82,83,84,85,86,87,88,89,90,91,92]. The direct carbonization of cellulose acetate membranes can form defects on the carbon matrix or even turn it into dust [31]. Therefore, there is a need to replace the acetyl group for a hydroxyl group. Some authors report the use of KOH and NaOH solutions to make the deacetylation procedure [91,92]. The solution can be aqueous, but it is more efficient when prepared in ethanol. The time of chemical exposure, the hydroxyl concentration, the content of the solution and the reaction time are very important parameters for this pre-treatment [31].

In 2011, He et al. prepared [76] carbon hollow fibers from cellulose acetate hollow fiber precursors that, before carbonization, were subjected to a deacetylation pre-treatment by immersion in a 0.075 M NaOH/96 vol.% ethanol solution. The influence of the deacetylation time (0.5, 1, 2, 4 and 8 h) on the membranes’ structure and their separation performance was assessed. Thermogravimetry indicates that the mass loss of the regenerated cellulose hollow fibers is larger for short deacetylation times, which indicates that most acetyl groups reacted with the NaOH solution within 2 h. The degradation of the precursor advances with the deacetylation reaction time and acetyl groups are progressively replaced by -OH groups. The highest permeability to CO_2_ was obtained for CMSM prepared from a cellulose acetate precursor after 2 h of deacetylation pre-treatment (220 barrer); the ideal selectivity of these membranes remained practically constant with the deacetylation times. The optimized CO_2_/CH_4_ and CO_2_/N_2_ selectivity obtained were, respectively, ~100 and ~30 [76] with θ values of 2.4 and 0.5, respectively.

A complementary study of the optimization of the deacetylation process conditions in sodium hydroxide ethanolic solutions was performed by He in 2017 [31]. The experimental variables considered were the concentrations of NaOH (0.05 M, 0.075 M and 0.1 M) and ethanol (96 vol.% and 50 vol.%) employed on preparing the NaOH solution; the time the fibers were placed in a 10 wt% glycerol bath (swelling time) and the deacetylation reaction time. The optimal deacetylation conditions were identified as consisting of a 0.075 M NaOH/96 vol.% ethanol solution, a swelling time of 24 h in glycerol and a reaction time of 2 h. The importance of the deacetylation parameters was ranked as follows: ethanol solution concentration > swelling time > reaction time > NaOH concentration [31].

The deacetylation process of cellulose acetate was also optimized in a pilot industrial scale production facility to produce hollow fibers [80]. Hollow fibers with the best mechanical properties and separation performances were obtained with 2.5 h of deacetylation at room temperature. The effect of other pre-treatments was also assessed, namely: (i) water washing; (ii) glycerol treatment concentration (5, 8, 10 and 15 vol% in water) to remove residual NMP; (iii) glucose washing with glucose playing the role of molecular spacer and (iv) fiber drying parameters (humidity, extra-load and temperature) [80]. The best permeation results after carbonization were obtained when the precursor fibers were washed from NMP using water at 30 °C for 3 h, followed by the immersion in a circulating glycerol solution with 5 vol% of glycerol. The permeability of CO_2_ of the resulting CMSM was >200 barrer with a CO_2_/N_2_ selectivity of 50 and θ = 0.3 for 5 vol% glycerol pre-treatment, compared to < 20 barrer, CO_2_/N_2_ selectivity of 40 and θ = 0.9 without pre-treatment [80]. Once again, the authors of this review suggest that glycerol is also acting as a molecular spacer. This glycerol pre-treatment increases the Robeson Index for the CO_2_/CH_4_ separation from 1 to 3.7.

Carbonized membranes after the deacetylation process exhibited low permeability to CO_2_ due to the high shrinkage and curliness. This problem was solved placing the deacetylated fibers in an aqueous solution of 7.5 wt% glucose. According to Haider et al., this glucose treatment preserves and protects the microporosity of the membranes produced on a pilot scale; glucose should then play the role of a molecular spacer, as described in Section 1.2. Finally, an overnight slow fiber drying was also found to create a final CMSM with higher permeability [80].

Rodrigues et al. [30], studying the preparation of CMSM from an optimized ionic liquid-regenerated cellulose precursor, immersed the precursor films in a softener bath with 5 wt% of propylene glycol for 1 min; the precursor films were then dried in an oven at 100 °C for 10 min. Propylene glycol was added to act as a molecular spacer, preserve the pores, reduce the warpage, improve the mechanical properties of the carbonized membranes and improve the membrane permeability [30].

Pre-treatments were also used in the case of microcrystalline cellulose membrane precursors processed from ionic liquids [73]. The produced fibers were immersed in a 10 wt% glycerol aqueous solution pre-treatment, before drying the fibers, to reduce the curl formation of cellulose hollow fibers during the drying process, in this case, 1 day at room temperature [73].

### 3.3. Effect of the Carbonization Conditions

The ideal CMSM should exhibit high permeability and selectivity, provided by a high micropore volume with a narrow pore size distribution [23]. A sequence of larger pores interconnected with narrower selective pores (constrictions) has to be present with a low tortuosity and porosity [29]. These characteristics are achieved during the core of the CMSM preparation process—the carbonization.

Carbonization is the process in which the polymer precursor is heated under controlled conditions, namely under an inert atmosphere, to produce a carbon membrane. As the temperature inside the furnace increases, the polymer matrix begins to decompose with a significant mass loss. During its decomposition, the release of heteroatoms occurs through the membrane by the flow of carrier gas used. Gases such as CO_2_, CO, H_2_, N_2_, H_2_O, HCN and NH_3_ can be released [93].

The release of heteroatoms creates different stresses and fragmentation on the membrane matrix and the polymeric chains entropy drives “plate” formation [26,93]. The reorganization of these “plates” allows the creation of the porous carbon structure formed by non-homogeneous graphene-like layers [26]. The voids between the non-homogeneous graphene-like layers are the micropores (responsible for the large permeance) and the slits between them are the constrictions, responsible for the molecular sieving (ultramicropores) [37]. On cooling the carbon membrane, the pores reorganize to form a cellular structure with a narrow and selective pore size distribution [93].

The pore structure is highly influenced by several carbonization variables. A small variation in one of these variables may cause a significant modification on the pore structure, and consequently, on the gas separation performance. The variables discussed below are the carbonization end temperature, the soak time, the carbonization gas atmosphere and its flow rate [37].

The carbonization end temperature must be higher than the decomposition temperature of the polymer precursor [94]. An increase in the carbonization end temperature results in a greater compaction of the membrane due to the inherent shrinkage and mass loss; this is accompanied by an increase in crystallinity and density, reducing the space between the graphitic layers. Therefore, typically, lower permeability and higher selectivity is achieved increasing the end carbonization temperature. However, the mechanical stability is also influenced by the carbonization end temperature [30,75], and CMSMs carbonized at higher temperatures are usually more brittle. Therefore, a compromise between membrane performance and mechanical stability should be attained.

The effect of the carbonization end temperature in cellulose-based CMSM has been addressed by various authors. Lie et al. optimized the carbonization protocol of a wood pulp precursor, hydrolyzed in TFA, studying the effect of the carbonization end temperature on the separation performance of the resultant membranes [89]. The membranes were carbonized in a vacuum atmosphere with a heating rate of 1 °C·min^−1^. Increasing the pyrolysis temperature up to 650 °C, the permeability of the tested gases increased. The permeability of H_2_ increased from 460 barrer (500 °C) to 1300 barrer (600 °C) due to pore opening. At 850 °C, the CMSM permeability decreased due the higher volumetric loss resulting from the greater stiffness of the membrane carbon structure [89]. A similar study with the same polymeric precursor was made by Grainger et al. [59] and the results obtained agree with these observations. The best performances were obtained with a CMSM carbonized at an end temperature of 650 °C, namely a permeability of H_2_ of 1388 barrer with a H_2_/CH_4_ ideal selectivity of 1157 displaying a Robeson Index of 79. In this work, a H_2_/CH_4_ ideal selectivity greater than 100,000 was also reported due to the non-permeance of CH_4_, reporting the highest θ for this separation.

Campo et al. [58], in 2010, and Rodrigues et al. [75], in 2019, both presented a study of the influence of the end carbonization temperature (in a range of 400 to 600 °C) on the performance of cellophane paper based CMSM. The permeability of all gases increased with the carbonization end temperature up to 550 °C, decreasing thereafter. The permeability increase observed up to 550 °C is related to the formation of the pore network. Above 550 °C the observed mass loss is minimal (thermogravimetric analysis) [75], as most of the volatiles have already been released. It is suggested that the reduction in permeability that occurs in membranes produced at 600 °C is due to a sintering mechanism forming a more rigid and constricted structure.

In 2019, the same authors prepared an ionic liquid-regenerated cellulose-based CMSM prepared at 550 °C and 600 °C [30]. These CMSM present a better permeability/selectivity balance when compared with the other cellulose-based CMSM with O_2_/N_2_ ideal selectivity well above the Robeson upper bound for the two temperatures. The CMSM carbonized at 600 °C presents a larger volume of micropores but with smaller length resulting in lower permeability than the CMSM prepared at 550 °C [30]. The permeability of CO_2_ decreased from 13 barrer to 4 barrer due to the tighter carbon structure induced by the sintering mechanism [30].

Some authors have introduced in the carbonization procedure dwells of 30 min [50,58,75]. These dwells avoid the rapid release of solvents and volatile matter at some temperatures preventing the formation of micro-cracks and defects on the carbon matrix.

The soak time corresponds to the time that the membranes are left at the final carbonization temperature. This time usually results in a better reorganization of the membrane pores, allowing to obtain more selective pores [95]. Therefore, this is a parameter that can also be adjusted to optimize the CMSM separation performance. Campo et al. [58] and Lie et al. [89] have introduced the soaking time on the production of cellulose-based CMSM. Longer soaking times were found to decrease the membrane microporosity, decreasing the permeability and increasing the membrane selectivity due to the sintering mechanism. Since then, several cellulose-based CMSM studies have presented a soaking time of 2 h in the carbonization step [31,73,77,80,81,89,96], see Table 4.

The choice of the carbonization atmosphere also affects the carbon structure due to the occurrence of different degradation mechanisms under different atmospheres. The carbonization can be made in a vacuum, inert gas atmosphere (He, N_2_ and Ar) or in an oxidative atmosphere (CO_2_). Hydrolyzed wood pulp membranes were carbonized in a vacuum and an argon atmosphere and it was concluded that the use of an inert gas causes an increase in membrane permeability [59] due to the increase in heat and mass transfer provided by the passage of the inert gas. By contrast, the carbonization under vacuum promotes the formation of denser carbon structures with smaller pore sizes and lower d-spacings [95].

Haider et al. [81] have analyzed the carbonization procedure under vacuum, N_2_ and CO_2_ atmospheres in a pilot-plant production of cellulose acetate based CMSM. The hollow fiber membranes carbonized under vacuum also presented low permeability compared to those produced under inert gas or oxidative atmosphere. The membranes carbonized under CO_2_ displayed higher CO_2_/CH_4_ permselectivities and a Robeson Index higher than 4, but had poorer mechanical properties [81]. The membranes prepared under N_2_ atmosphere exhibited higher separation performance with a permeability of CO_2_ of 410 barrer [81] and a θ of ~2. The N_2_ flow accelerated the carbonization creating a more open porous structure.

Helium was used by Sazali et al. [97] as an inert atmosphere to carbonize P-84 co-polyimide/nanocrystalline cellulose membrane. The permeance to hydrogen of the carbonized membrane was higher compared with the permeance to hydrogen of the same precursor carbonized under N_2_ or Ar atmospheres.

The gas flow rate used during carbonization process is also an important parameter that must be taken into account. In cellulose-based CMSMs, flow rates between 80–170 mL⋅min^−1^ [30,58,73,75] have been typically used. Some studies indicate that the use of higher flow rates produce membranes with higher microporosity and higher permeability without impairing the selectivity [37].

The heating rate during the carbonization step can change the release rate of volatiles, changing the membrane structure. If high heating rates are used, the release of residuals will cause cracks or holes in the membrane structure. By contrast, low heating rates tend to increase the crystallinity of the carbon matrix lowering permeability [94]. This parameter requires extensive and careful optimization. Many authors used various heating rates in the manufacture of CMSM, usually between 0.1 and 13 °C·min^−1^ [97]. The heating rate effect in cellulose-based CMSM was studied by Sazali et al. [97,98,99]—heating rates of 1, 3, 5 and 7 °C·min^−1^ were tested and the optimized condition found was 3 °C·min^−1^.

He et al. studied the effect of single carbonization steps into the membranes transport. They studied the influence of the gas atmosphere (vacuum, N_2_ or CO_2_), heating rate (1, 2 or 4 °C·min^−1^), final soak time (0, 2 or 4 h) and the final temperature (550, 650 or 750 °C) on the production of deacetylated cellulose acetate-based hollow fiber CMSM. The authors ranked the order of importance of the studied factors as: gas atmosphere > carbonization end temperature > heating rate > soak time. The optimum carbonization procedure for CO_2_/CH_4_ separation was under CO_2_ atmosphere at 550 °C, with a heating rate of 4 °C·min^−1^ and 2 h of soak time [77].

### 3.4. Effect of the Post-Treatments

At the end of the carbonization process, the pore network structure is already formed and prepared for the molecular sieving gas separation. However, some post-treatments can still be applied to repair defects or cracks formed in the pyrolysis reaction, to open or narrow the pore size distribution and to passivate the inner surface towards the oxygen chemisorption. These include coating, post-pyrolysis, oxidation, activation, passivation and chemical vapor deposition (CVD) [23].

When the prepared CMSMs present cracks and defects, a coating with another polymer can be applied followed by a new carbonization step [40]. The post-pyrolysis reaction causes a decrease in the pore volume that can result in a decrease of the CMSM separation performance [37].

Post-oxidation treatments can be used to open the pore structure of the CMSM. The post-oxidation treatment can reopen or enlarge the carbon membrane pores [29]. This post-treatment can be performed with pure oxygen, oxygen mixed with other gases, air, steam, carbon dioxide, chlorine and nitrogen oxides and oxidizing agents such as peroxide, nitric acid and others [23]. The oxidation can be processed at different activation temperatures and dwells to obtain the required pore structure. As the oxidation temperature increases, an increase in the pore length occurs, thus allowing an overall permeability increase but a reduction of the ideal selectivity [100,101,102]. Koros et al. developed a low temperature oxidation method to open pores [103], and more recently, a new oxidation method named Dual Temperature Secondary Oxygen Doping, where a small amount of oxygen at high temperature is used after the carbonization to enhance de O_2_/N_2_ separation maintaining the membrane permeance [104].

A different post-treatment is passivation, which consists of placing the CMSM under a H_2_ atmosphere [82] to remove some of the oxygenated functional groups or chemisorbed oxygen molecules, stabilizing the surface of the membrane and weakening its hydrophilic character [29].

In 1997, Soffer et al. [105] patented a chemical vapor deposition (CVD) post-treatment method to improve the membrane selectivity. With the CVD technique, narrower pore size distributions can be obtained through the pyrolytic decomposition of organic species, such as propane, propylene, ethylene or benzene, that are introduced into the porous structure of the CMSM. These organic species should have high chemical stability, an adequate reactivity to absorb on the porous surface and not produce intermediate species on degradation [37]. The excess of vapor and non-optimized conditions such as temperature, flow rate, time and composition can block the pore network, destroying the membrane’s ability of molecular sieving. The CVD on carbon membranes can produce three types of deposition: homogeneous, in-layer or ad-layer [105]. The homogeneous deposition can form a narrow and desired pore size distribution.

The processes patented by Soffer et al. were acquired by Carbon Membranes Lda., which introduced the CVD technique on the cellulose-based hollow fiber membranes using propylene as a carbon source [70]. In this patent, CVD is applied on the bore side of the hollow fiber (in-layer deposition). After CVD, the membrane permeability is reduced, and therefore, a further step was suggested, called activation [105]. This activation step, carried out in an oxidant atmosphere at high temperatures, opens pores selectively forming a well-defined pore structure on the CVD layer, with a very narrow pore size distribution [70]. For example, the CMSM permeability of N_2_ before CVD and activation processes was ~6 barrer and it increased to ~300 barrer with CVD/activation treatments [70]. Furthermore, the CMSM remained impermeable to SF_6_ demonstrating that no cracks or defects were produced.

Haider et al. [7] used the method of successive CVD with propylene and oxidation and reduction steps proposed by Soffer [105] and developed at Carbon Membranes Lda., to prepare a tailored pore structure in the CMSM hollow fibers from deacetylated cellulose acetate. The original untreated CMSMs displayed a reduced permeability of CO_2_ and a low CO_2_/N_2_ selectivity. The post-treatment consisted of the following steps [7]: (i) heating to 300 °C under synthetic air at 4 °C·min^−1^; (ii) a dwell under 20% of oxygen for allowing the post-oxidation; (iii) a second heating with N_2_ until 500 °C at 4 °C·min^−1^; (iv) feed of the reducing agent, H_2_, at 500 °C; (v) CVD treatment at 500 °C with propylene for a short period of time; (vi) cooling under N_2_ atmosphere until 300 °C. Repeat steps (ii), (iii) and (iv) to open and refine the pore structure made with the CVD coating, as described by Soffer [105]. The results obtained showed that this post-treatment increased the permeability of CO_2_ 50,000 times and the CO_2_/N_2_ selectivity 41 times; when compared with untreated membranes, the CMSM permeability of CO_2_ increase from 0.006 to 300 [7]. The non-CVD CMSM displayed a θ lower than 1 and the CMSM post-treated presented a θ higher than 6.

### 3.5. Effects of Inorganic Filler Addition and Polymer Blending

Some authors have investigated the effect that the addition of inorganic fillers to the polymeric precursors has on the final structure and performance of the CMSM. Mesoporous silica [106] and boehmite nanoparticles [48,49] are examples of inorganic fillers loaded to polymeric precursors used in the production of CMSMs. The addition of a thermally stable element to the precursor can improve the membranes permeability and selectivity to selected gas species [48], especially if the additive has good chemical affinity with that particular gas. This strategy has been used, for example, to improve the permeability of H_2_, with the addition of Pd or Pt due their affinity towards hydrogen [107].

Teixeira et al. [108] produced CMSM containing two different nanofillers: alumina and silver. The Ag doping improved the membrane performance, increasing the propylene/propane selectivity from 15 to 38. When metal nitrates were used, the release of the nitrate ion during the carbonization acted as a porogenic agent increasing the pore volume.

Studies of addition of inorganic fillers to cellulose-based CMSMs were carried out by Lie and Hagg [57]. These authors added metals to the wood pulp: oxides such as Ca, Mg and Fe (III) and nitrates such as Ag, Cu, Fe (III). In this work, the addition to the precursor of oxide metals increased the micropore volume of the resulting CMSM, as the metal acted as a spacer [109]. The addition of metal nitrates had a different effect on the separation performance as it reduced significantly the permeability of CH_4_ but not of other gases such as H_2_ and CO_2_. Lie and Hagg developed a simple and fast method for regenerating cellulose-based CMSMs [89]. They doped the membranes with iron and applied a low voltage direct electric current (10 mA; 17.5 V) to the permeation module. This current promotes gas desorption, increasing the membrane permeability–regeneration, due to ohmic heating. The addition of 1.8 wt% iron increased the permeability of the membrane to O_2_ from 54 to 86 barrer and to CO_2_ from 190 to 310 barrer, improving the selectivity of the CO_2_/CH_4_ membrane from 41.3 to 147.6 [89] increasing the θ from 0.85 to 3.64. The increase in the concentration of iron caused the gas separation properties to deteriorate, with a significant reduction in permeability.

The purification of H_2_ from a mixture with CH_4_ was studied by the same group [59]. They obtained a permeability to H_2_ greater than 1388 barrer and a H_2_/CH_4_ selectivity greater than 100,000. The addition of copper nitrate (II) to the precursor (0–6 wt%) was also studied and resulted in an increase of H_2_/CH_4_ selectivity, due to a contraction in the pore size. The addition of 6 wt% of copper nitrate caused the appearance of a surface layer and a consequent decrease in the permeability of H_2_.

Some authors studied mixed matrix membranes of polymer blends, as precursors for CMSM [110,111]. This strategy can be used to improve the separation performance and the mechanical properties, or even reduce the aging and the fabrication cost of the polymer-based carbon membranes [112]. Allowing one polymer to thermally decompose first (thermally unstable polymer, i.e., pyrolyzing polymer) can create different porous structures [23], acting as a molecular spacer.

The molecular spacer strategy has also been used by the NTNU membrane research group, who have incorporated, since 2011, 5 wt% of PVP to their cellulose acetate precursor solutions [76,79,80,90] for increasing the porosity of the resulting CMSM.

Nanocrystalline cellulose (NCC) synthesized from recycled newspaper was blended as an additive with the polyimide precursor BTDA-TDI/MDI (P-84). The NCC acts as a pore forming agent due to its lower decomposition temperature compared to polyimide (PI) [84,85,113]. First, pore formation occurs due to the decomposition of the NCC and only later the pore network of the polymer in larger concentration begins to form. Different NCC loadings, several heating rates [97], different carbonization temperatures and different pyrolysis atmosphere [86] were tested.

### 3.6. Aging and Regeneration of Cellulose-based CMSM

One of the greatest challenges for the commercialization and industrial use of CMSMs is their aging when exposed to organic contaminants, humidity and oxygen namely from air [37,71,114]; in fact, in general CMSM do not exhibit constant permeability performance over a long period of time, due to the aging effects. This aging can be reversible or irreversible, and several research groups have investigated various methods of passivation and regeneration for this type of membranes. The regeneration methods studied for CMSM regeneration can be thermal, chemical, electrochemical, ultrasonic or with microwaves. They can be operated online or offline and their use depends on their complexity and energy demand [114].

#### 3.6.1. Exposure to Humidity

**Figure 10:**
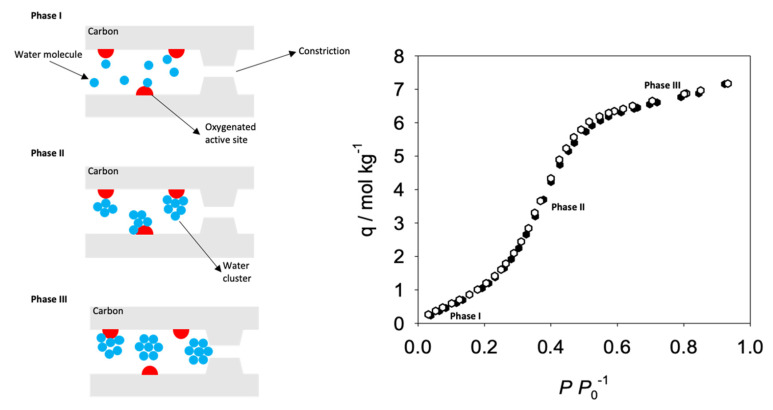
Water vapor adsorption mechanism in CMSM. Water vapor isotherm adsorption of a cellulose-based CMSM (adapted with permission from [116]).

In 1995, Jones et al. [115] found that the CMSM separation performance was drastically reduced when exposed to 85% relative humidity (RH). Years later, Lagorsse et al. [71] studied the aging of cellulose-based CMSM from Carbon Membranes Lda. A decrease of 50% on the membrane performance (permeance to CO_2_ at 305 K) was observed when CMSMs were exposed to 32.5% RH. The same authors observed that water adsorption isotherms display type V behavior and a desorption hysteresis, see Figure 10; based on these observations they proposed a water-induced aging mechanism on cellulose-based CMSM [82,116]. The water vapor adsorption mechanism occurs in three phases. Firstly, water adsorbs onto hydrophilic sites (Phase I); the adsorbate-adsorbate interaction promotes the adsorption of further water molecules via hydrogen bonding generating a water cluster (Phase II); the resulting water cluster gains enough energy to release from the hydrophilic site rolling until blocking a constriction (Phase III)—reducing the separation performance [71,116].

In 2019, Rodrigues et al. [75], performed permeation experiments in the presence of 75–77% RH using a cellophane paper based CMSM carbonized at 550 °C. The results with humidified steams showed an increase in the CMSM permeability (due the very fast water vapor permeation) concluding that humidity does not affect the membrane’s ability to permeate and separate gases. Water vapor isotherms at 25 °C were performed to understand the water vapor stability. As the carbonization end temperature increased from 400 to 550 °C, the water vapor adsorption isotherms became increasingly linear, indicating an increasingly higher hydrophilic character. At higher temperatures the isotherms started curving again, displaying a marked S-shape as shown in Figure 10. For the first time, a linear water vapor adsorption isotherm was reported for CMSM. The linearity is characteristic of carbon materials with hydrophilic sites homogeneously distributed throughout their inner surfaces, allowing water molecules to jump smoothly between polar sites and avoiding the formation of water molecule clusters. X-ray photoelectron spectroscopy (XPS) and Inductively Coupled Plasma (ICP) analysis were performed, and both techniques indicated the presence of metallic and semi-metallic elements (ionic sodium and silica nanoparticles) on the CMSM surface. The same hydrophilic behavior was observed in ionic liquid-regenerated cellulose-based CMSM. The results of the permeation of humidified steams do not evidence any sort of aging effect in the CMSM [30].

#### 3.6.2. Exposure to Oxygen and Organics

Frequently, when CMSMs are exposed to air, irreversible oxygen chemisorption can occur. Oxygen chemisorbs at active sites on the membrane carbon surface. The C-O complexes are formed in the inner structure, causing a decrease on the porosity and promoting a significant permeability loss [117]. Lagorsse [71] reported that CMSM made from a cellulose precursor lost 50% of N_2_ permeance when exposed to long term ambient air, due to oxygen chemisorption. Oxygen adsorbs on the membrane surface, promoting the formation of oxygen groups on the surface [114].

CMSMs made from cellophane paper are also susceptible to O_2_ chemisorption when exposed to ambient air over several days [75]. The CMSM permeability to O_2_ and N_2_ was evaluated over 2 to 7 days. Although the CMSM performance decreased almost 70% due to O_2_ chemisorption, this process (O_2_ chemisorption) favored the ideal membrane selectivity (CMSM produced at 600 °C presents a O_2_/N_2_ ideal selectivity of >800) due to the larger reduction observed on the permeability of N_2_ (<0.001 barrer). To the best of the authors’ knowledge, these CMSM displayed the highest Robeson Index values for the different gas separations.

The first passivation method reported for preventing the oxygen chemisorption uses H_2_ at high temperatures for treating the CMSMs [118,119]. This method, however, proved to be of low efficiency and new approaches have since been proposed and assessed [71]. Menendez and Fuertes reported that the exposure of phenolic resin based CMSMs to propylene can prevent the oxygen chemisorption [117]. These authors based their study on the method of chemical regeneration with propylene discovered first by Jones and Koros. In this later case, propylene acted as a cleaning agent, removing surface contaminants and active sites on the carbon matrix [120].

The propylene passivation method (2 bar and room temperature for 10 days) was successfully used by Rodrigues et al. [75] to stabilize cellophane-based CMSM against oxygen chemisorption. Haider et al. [96]. have also used propylene to prevent the oxygen/hydrocarbons chemisorption on deacetylated cellulose acetate based CMSM. Propylene was proposed to act in two different ways on the carbon membrane, increasing the membrane permeability. First, as a solvent for the adsorbed components, removing them (functioning as a cleaning agent); second, causing pore expansion due to the electronic repulsion between the propylene π cloud and the π cloud of the graphene layers that make up the micropore [96]. In this work, the CMSM performance was studied over 5 months under air, H_2_S, n-hexane, CO_2_ and methane environments [96]. At this point, it is worth noting that in a recent study of cellulose-based CMS adsorbents, Andrade et al. [121] proposed that propylene removes functional groups that are prone to chemisorb O_2_.

CMSM have a very high affinity to organic compounds [37] and the exposure to these organics blocks the membrane microporous structure impairing its separation performance [120]. A heat treatment, up to 80 °C, was tested to regenerate the CMSM, but this was not very effective [96]. Electrical regeneration proved to be the most promising regenerative method [96].

An on-line electrical current was applied to prevent the active sites on the carbon surface from reacting with O_2_, while the membrane was in operation. In this case, there was a slight decrease in membrane permeability and an increase in selectivity [96]. This electrical method for regenerating carbon membranes made from cellulose was discovered by Lie and Hagg in 2005 [57]. This method is based on applying a low voltage direct current to iron doped carbon membranes. This applied current causes the rate of gas desorption to increase due to ohmic heating [57].

One of the target separations for CMSM is the CO_2_ removal from the natural gas/biogas. Haider et al. [122] found that when the cellulose-based CMSMs were exposed to concentrations of H_2_S from biogas, they lose their ability to permeate CO_2_, due to the worsening of aging. Therefore, an additional step of H_2_S removal from the natural gas/biogas was shown to be necessary before CO_2_ removal using a CMSM.

## 4. Principal Applications of Cellulose-based Carbon Molecular Sieve Membranes

CMSMs, due to their high separation performance and high thermal and chemical stability, are the most promising type of gas separation membranes for corrosive environments.

A key factor for the commercialization of CMSMs is their manufacturing cost and cellulose is an inexpensive material that makes cellulose-based CMSMs potentially more attractive. Furthermore, as reported in this review, the aging that usually affects CMSMs reduces their performance, rendering them commercially uninteresting. However, in cellulose-based CMSM, this problem has already been overcome, with high performances, making these membranes increasingly closer to the market [75,122].

Cellulose-based CMSM have several industrial applications such as nitrogen generation and oxygen enrichment from O_2_/N_2_ separation, the separation of hydrogen from hydrocarbons for the recovery of hydrogen in refineries, the separation of CO_2_ from methane for the treatment of biogas or natural gas and the dehydration of currents (vapor/gas separation) or the separation of light hydrocarbons [123]. Xenon recovery from anesthetic cycles has also been studied as an application of carbon membranes made of cellulose [124].

Carbon Membranes Ltd., in Israel, and Blue Membranes GmbH, in Germany, were the first two companies to commercialize CMSM. However, these two companies were unable to overcome the main CMSM challenges presented in this review. MemfoACT AS [17] is another company that produced cellulose acetate-based hollow fiber CMSMs. This company was founded in 2008 by May-Britt Hagg, Jon Lie and Arne Linderbrathen, and its produced membranes targeted, as their core business, the separation of the gas mixtures CO_2_/CH_4_, H_2_/CH_4_ and O_2_/N_2_. The separation performance of CMSM from MemfoACT AS presented a O_2_ permeability of ~12 barrer with a O_2_/N_2_ selectivity of ~17, corresponding to a Robeson Index of 2.2, and presented a CO_2_ permeability of 154 barrer with a CO_2_/CH_4_ selectivity of 245 (Robeson Index of 4.6) [17]. This company had a total capacity production plant of 700 m^2^/year, which was equivalent to 250 modules. MemfoACT installed more than 200 plants for CO_2_ removal; however, unfortunately, it closed in 2015 [122].

### 4.1. O_2_/N_2_ Separation

Ambient air consists mostly of a mixture of oxygen (~21%) and nitrogen (~79%) and the separation of these two components is highly needed for several applications. Most of the studies in membranes for gas separation technology focus on this separation [114]. Nitrogen and oxygen markets require often product streams with concentrations of 99% or higher [125]. Nowadays, the separation of air is mostly performed by cryogenic distillation or by pressure swing adsorption, but these techniques are energy-intensive, and therefore, there is a growing interest in the use of membrane separation technology [126].

**Figure 11:**
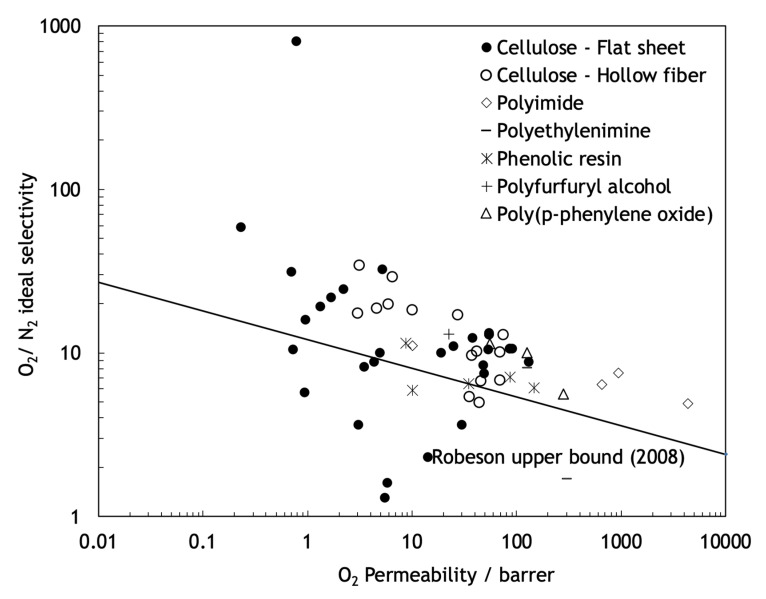
Separation performance of CMSM with different polymeric precursors for O_2_/N_2_ separation.

Polymeric membranes, which operate through a sorption-diffusion mechanism, exhibit relatively low O_2_/N_2_ selectivity, which makes them uninteresting for this separation. By contrast, CMSM display higher selectivity with high permeability to oxygen (Figure 11), due to the molecular sieving mechanism. The lightness and compactness of the CMSM modules are an additional advantage for this separation [7].

Although the better performance of CMSM for the O_2_/N_2_ separation is observed with different polymeric precursors, the cellulose-based CMSMs present the most favorable balance between permeability and selectivity compared to phenolic resin [22,50,108,127], polyimide [22,128,129,130,131], PEI [56,132,133], Polyfurfuryl alcohol (PFA) [134,135] and Poly(p-phenylene oxide) (PPO) [111,136,137] precursors (Figure 11). The polyimide-based CMSMs present higher permeability but their selectivity are lower and its precursor cost makes their membranes less competitive in the market [31].

Compared with the other polymeric precursors, cellulose-based CMSM have greater separation factors, as can be seen in Figure 11. Cellulose-based CMSM present the record values of Robeson Index for the O_2_/N_2_ separation (θ = 63).

### 4.2. CO_2_/CH_4_ Separation

Carbon membranes can be used for removing CO_2_ to purify methane produced from different sources [23]. Natural gas sweetening is the largest growing market for CO_2_/CH_4_ membrane separation [7]. Biogas has been used to reduce greenhouse gas emissions [138]. Untreated natural gas contains methane light and heavy hydrocarbons, water, H_2_S and CO_2_. The sweetening of natural gas is also applied to biogas to purify methane by removing carbon dioxide and the acid gases. The conventional separation method of CO_2_ from the natural gas/biogas is based on an absorption process driven using amines [139] or, more recently, using amino acids [140], energy intensive processes and with environmental problems [114]. Therefore, membrane separation has been studied as an alternative. Several studies tested polymeric membranes, but their low CO_2_/CH_4_ selectivity, due to the plasticization and compaction effect of the membrane, makes these membranes not competitive for this market [141]. In contrast, carbon membranes have high permeability of CO_2_ and high CO_2_/CH_4_ selectivity and are able to operate at high pressure and high temperatures, being, therefore, competitive for natural gas sweetening.

**Figure 12:**
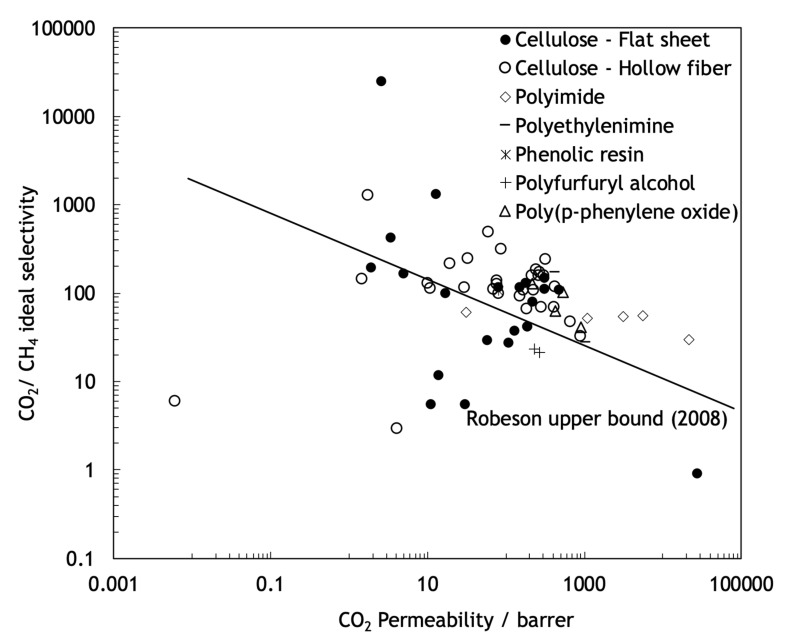
Separation performance of CMSM with different polymeric precursors for CO_2_/CH_4_ separation.

Cellulose-based CMSM present themselves as the strongest candidates for this multi-million-dollar market due their excellent balance between CO_2_ permeability and CO_2_/CH_4_ ideal selectivity (over 100) (Figure 12). He et al. simulated a biogas plant with a two-stage cellulose acetate based CMSM with a CO_2_/CH_4_ selectivity of 60 at 20 bar [138]. The lowest biogas upgrading cost of 0.078 $·m^−3^ was achieved for a 1000 m^3^(STP)·h^−1^ biogas plant at 8.5 bar of feed pressure. A processing natural gas cost of 0.011 $·m^−3^ was achieved at 90 bar of feed pressure for a two-stage cellulose acetate-based CMSM with a recycling in the second stage to achieve >98% CH_4_ with a <2% loss of CH_4_ [8]. Haider et al. found that the CO_2_/CH_4_ selectivity of cellulose acetate-based CMSM remains constant when the feed pressure is increased up to 70 bar, but the CO_2_ permeability decrease 50% when the temperature reaches 120 °C. The simulation results showed that a single-stage design can separate streams with up to 50% CO_2_ with CH_4_ recovery greater than 97.7% with a total cost of ~300 $·Nm^−3^ of feed gas [7], i.e., a cost three times lower than with polymeric cellulose acetate and polyimide membranes.

Once again, cellulose-based CMSM have the highest reported Robeson Index value (100) for this separation. This value is much higher than that exhibited by CMSM based on other polymeric precursors.

Cellulose-based carbon membranes were used for biogas upgrading from a biogas stream proven by the anaerobic digestion of residuals to be applied in vehicle fuel (methane required purity: 96–98 vol%) [10]. The operation was carried out in a pilot unit for 8 h. The authors recovered 98% of methane in 5 h with a purity of 96%.

### 4.3. Hydrogen Recovery

**Figure 13:**
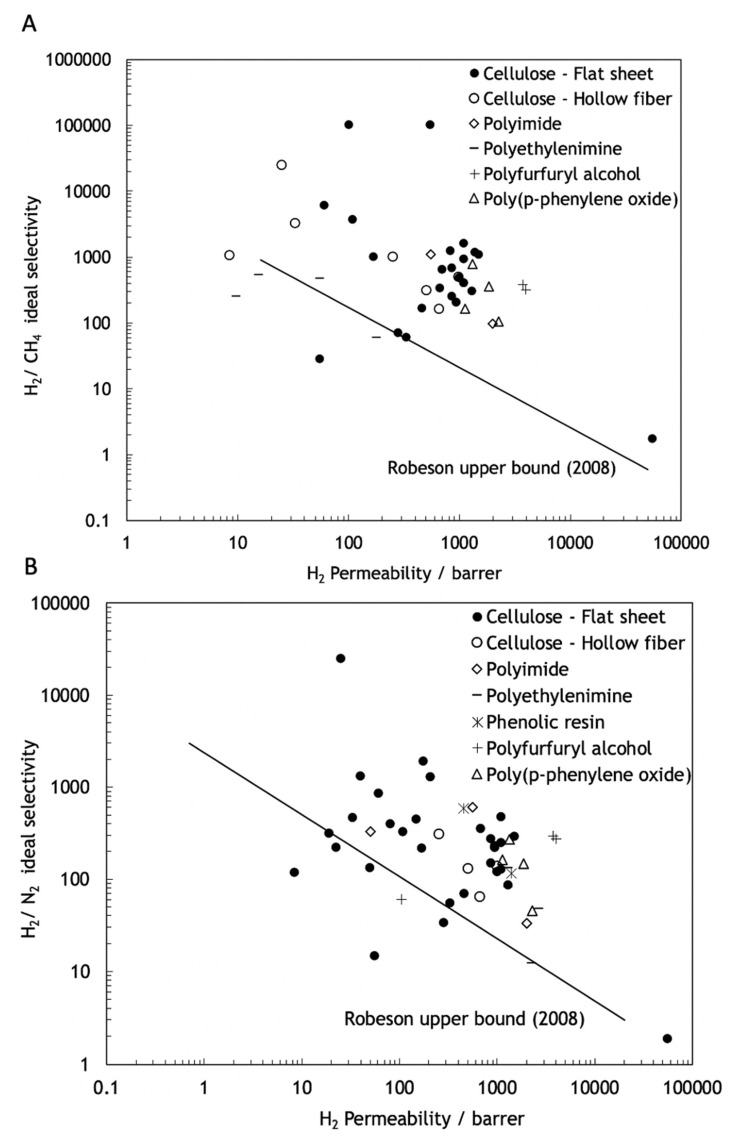
Separation performance of CMSM with different polymeric precursors for: (**A**) H_2_/CH_4_ separation; (**B**) H_2_/N_2_ separation.

For producing fuel cells grade hydrogen, membranes compete with cryogenic separation and pressure swing adsorption processes for energy efficiency, cost and being environmentally friendly [12]. Once again, polymeric membranes are not competitive for this market as they require high recompression costs [142]. The CMSM presents higher hydrogen selectivity making the gas compression costs lower. Cellulose-based CMSM present higher H_2_/X selectivity when compared with CMSMs based in different polymeric precursors (Figure 13) and these, combined with their low cost, high energy efficiency and non-aging properties in harsh environments, confers a high industrial potential. The cellulose-based CMSM presents the highest Robeson Index values for these two separations. Although the carbon membranes made from PFA and PPO precursors have much higher permeability values to hydrogen, their Robeson Index values are very close to those recorded for membranes made by cellulosic precursors.

In Table 5 the record Robeson Index values are presented for the four target separations analyzed above.

## 5. Conclusions and Future Directions

Cellulose-based CMSMs have high potential for separating O_2_/N_2_ and CO_2_/CH_4_ mixtures and purifying H_2_. However, before entering into industrial production the numerous fabrication parameters still need to be optimized and their performance further improved. This will also require a better understanding of processing-structure-performance relationships in this particular type of CMSM.

The effect of the different type of cellulose precursors on the final CMSM properties is still poorly understood and needs further investigation. This includes, for example, achieving a better understanding of the effect of cellulose molecular mass (MM) and polydispersity (PDI) on the final membrane properties. It also involves studying the effect of the dissolution system (ionic liquids, NMMO, among others) and the precursor solutions need to be optimized concerning several parameters such as cellulose concentrations and the addition of molecular spacers (plasticizers, additives) with different concentrations. Experimental carbonization conditions such as the heating rate and flowrate of the inert gas should be carefully studied and optimized, as well as post-treatments (CVD) used during the membrane fabrication process.

In the case of precursor membranes using molecular spacers (with different boiling or decomposition temperatures), the effect of the temperature history during carbonization on the final CMSM properties is still poorly understood. The introduction of some temperature plateau below the boiling temperature of the molecular spacer may eventually solve this problem by slowing down the evaporation. For example, if the molecular spacer evaporates too quickly, this may cause oversized pores. Therefore, the temperature history should be optimized individually for each polymer precursor-molecular spacer system. Additionally, the mechanism of pore formation by molecular spacers is still poorly understood, as it involves a complex interplay between several variables, including: (i) the molecular spacer boiling/degradation temperature and polymer degradation temperature; (ii) the kinetics of molecular spacer evaporation/volatilization; (iii) the type of phase behaviour in the binary system “polymer precursor-molecular spacer” (upper or lower critical solubility temperature—UCST, LCST—or a combination of both) and the location of the system in the one- or two-phase region, as this will determine if the molecular spacer is dispersed or agglomerated in the polymer precursor matrix as the temperature increases; (iv) the type of phase separation mechanism (spinodal decomposition or nucleation and growth), which can create very different morphologies, among other factors.

Finally, the Robeson Index, suggested for the first time in this article, proved to be a quite instructive tool for comparing the performance of a given membrane with the Robeson upper bound.

## Figures and Tables

**Table 1:** Values of the front factor and the upper bound slope for the different gas pair separations. (adapted from [14,15]).

$\alpha_{ij}$	*k*	*n*
O_2_/N_2_	1,396,000	−5.666
He/H_2_	59,910	−4.864
CO_2_/CH_4_	5,369,140	−2.636
CO_2_/N_2_	30,967,000	−2.888
H_2_/CO_2_	4515	−2.302
He/O_2_	4600	−1.295
H_2_/N_2_	97,650	−1.484
He/CO_2_	3760	−1.192
H_2_/O_2_	35,760	−2.277
H_2_/CH_4_	27,200	−1.107
He/N_2_	19,890	−1.017
He/CH_4_	19,800	−0.809

**Table 2:** Values of the y-intercept of the Robeson upper bound ($\beta =k^{-1/n}$) for the different gas pair separations.

$\alpha_{i,j}$	O_2_/N_2_	He/H_2_	CO_2_/CH_4_	CO_2_/N_2_	H_2_/CO_2_	He/O_2_	H_2_/N_2_	He/CO_2_	H_2_/O_2_	H_2_/CH_4_	He/N_2_	He/CH_4_
**β**	12.15	9.599	357.3	392.5	38.69	673.6	2302	998.4	99.94	10,138	16,857	204,683

**Table 3:** Gas separation results of different cellulosic precursor based CMSM.

Cellulosic Precursor	Conditions ^1^	Permeability (Barrer)	Ideal Selectivity	Robeson Index	Ref.
**Cellulose Cuprammonium**	Not available Hollow fiber	O_2_: 34.9 CO_2_: 183.2	O_2_/N_2_: 5.4 CO_2_/N_2_: 28.3	0.83	[70]
**Wood Pulp**	1 wt% TFA *T*_carbonization_ = 550 °C Soak time = 2 h Flat membrane	O_2_: 54 CO_2_: 190	O_2_/N_2_: 13.2 CO_2_/N_2_: 46.3 CO_2_/CH_4_: 41.3	2.20 0.05 0.85	[57]
	70% DMSO/30% EmimAc *T*_carbonization_ = 550 °C Flat membrane 5 wt% of MPG	O_2_: 5.16 CO_2_: 13.4	O_2_/N_2_: 32.3 CO_2_/N_2_: 83.8	3.55 0.52	[30]
**Cellulose Acetate ^2^**	100% NMP *T*_carbonization_ = 550 °C Soak time = 2 h Hollow fiber	O_2_: 41 CO_2_: 164	O_2_/N_2_: 10.3 CO_2_/N_2_: 40 CO_2/_CH_4_: 109.3	1.63 0.60 2.12	[77]
	100% NMP *T*_carbonization_ = 650 °C Hollow fiber	CO_2_: <20	CO_2_/N_2_: 40 CO_2_/CH_4_: 120	0.29 1.05	[80]
	100% NMP *T*_carbonization_ = 650 °C Hollow fiber 7.5 wt% Glycerol 7.5 wt% Glucose	CO_2_: 260	CO_2_/N_2_: 53.3 CO_2_/CH_4_: 160	0.93 3.69	[80]
	100% NMP *T*_carbonization_ = 650 °C Hollow fiber Oxidation/Reduction/CVD	CO_2_: 318	CO_2_/N_2_: 82 CO_2_/CH_4_: 246	1.54 6.13	[7]
**Cellophane Paper**	*T*_carbonization_ = 550 °C Flat membrane	O_2_: 4.33 CO_2_: 16.9	O_2_/N_2_: 10.3 CO_2_/N_2_: 51.2	0.94 0.35	[58]
	*T*_carbonization_ = 550 °C Flat membrane Passivation with C_3_H_6_	O_2_: 2.75	O_2_/N_2_: 8.1	0.80	[75]
	*T*_carbonization_ = 600 °C Flat membrane 4 days of air exposure	O_2_: 0.78 CO_2_: 2.57	O_2_/N_2_: 800 CO_2_/N_2_: 2600 CO_2_/CH_4_: 2600	63.0 9.18 100	[75]
**Microcrystalline Cellulose**	75% DMSO/25% EMIMAc *T*_carbonization_ = 600 °C Soak time = 2 h Hollow fiber 10 wt% Glycerol	O_2_: 74 CO_2_: 239	O_2_/N_2_: 13 CO_2_/N_2_: 4.5 CO_2_/CH_4_: 186	2.29 0.08 4.16	[73]

^1.^ (1) Solvent; (2) Carbonization end temperature; (3) Soak time; (4) Membrane configuration; (5) Precursor/CMSM treatment. ^2.^ Cellulose acetate is blended with PVP (22.5% CA /5% PVP/72.5% NMP).

**Table 4:** Influence of the soak time on cellulose-based CMSM carbonized at 550 °C.

Precursor	Soak Time (h)	Permeability (Barrer)		Ideal Selectivity		Robeson Index	
		O_2_	CO_2_	O_2_/N_2_	CO_2_/CH_4_	O_2_/N_2_	CO_2_/CH_4_
**Cellophane Paper**	0	4.33	16.9	8.80		0.94	
	1	4.87	17.0	9.90	98.1	1.08	0.80
	4	1.67	5.00	21.7	166.7	1.96	0.86
	8	0.70	1.93	31.2	193.0	2.41	0.69
**Wood Pulp**	0	90	310	10.5	110.7	1.91	2.73
	2	54	190	12.9	41.3	2.15	0.85

**Table 5:** Higher reported Robeson Index values for the O_2_/N_2_, CO_2_/CH_4_, H_2_/CH_4_ and H_2_/N_2_ separations for different CMSM polymeric precursors (Please find the other results in the Table S1 in the Supporting Information).

	O_2_/N_2_	CO_2_/CH_4_	H_2_/CH_4_	H_2_/N_2_
**Cellulose—Flat Sheet**	63	100	2943	94.8
**Cellulose—Hollow Fiber**	42	6.6	24.3	6.3
**Polyimide (PI)**	2.0	18.6	31.8	18.6
**Polyethylenimine (PEI)**	1.6	4.9	1.7	7.3
**Phenolic Resin (PR)**	1.5	1.6	-	15.6
**Polyfurfuryl Alcohol (PFA)**	1.9	0.5	62.3	32.6
**Poly(p-phenylene Oxide) (PPO)**	1.9	3.1	50	15

## Supplementary Materials

The following are available online at https://www.mdpi.com/1420-3049/25/15/3532/s1, Table S1: Summary table with the separation performances of all cellulose-based CMSM.
