# Supplementary material for: Cellulose-Based Carbon Molecular Sieve Membranes for Gas Separation: A Review
Source: Molecules. 2020 Aug 1;25(15):3532. doi: 10.3390/molecules25153532 (PMC7435847; doi:10.3390/molecules25153532)
Supplement: Supplementary file 1 [file molecules-25-03532-s001.docx]

Cellulose based carbon molecular sieve membranes for gas separation: a review

Tiago Araújo^1^, Gabriel Bernardo ^1^ and Adélio Mendes ^1,^*

^1^ LEPABE - Laboratory for Process Engineering, Environment, Biotechnology and Energy, Faculty of Engineering, University of Porto, Rua Dr. Roberto Frias, 4200-465 Porto, Portugal

***** Correspondence: [mendes@fe.up.pt](mailto:mendes@fe.up.pt)

**Supporting Information**

Table S1. Summary table with the separation performances of all cellulose based CMSM.

|  | Percursor | T_carbonization_  (ºC) | T_test_  (ºC) | P_feed_  (bar) | Permeability (barrer) | | | | | | Ideal Selectivity | | | | | *Robeson Index* | | | | | Reference |
| --- | --- | --- | --- | --- | --- | --- | --- | --- | --- | --- | --- | --- | --- | --- | --- | --- | --- | --- | --- | --- | --- |
|  |  |  |  |  | He | H_2_ | CO_2_ | O_2_ | N_2_ | CH_4_ | O_2_/N_2_ | CO_2_/CH_4_ | CO_2_/N_2_ | H_2_/CH_4_ | H_2_/N_2_ | O_2_/N_2_ | CO_2_/CH_4_ | CO_2_/N_2_ | H_2_/CH_4_ | H_2_/N_2_ |  |
| 1a | Cellophane paper (CP) | 400 | 29.5 | 2 | 19.3 | 22.2 | 3.93 | 0.93 | 0.10 |  | 5.7 |  | 39.3 |  | 222 | 0.46 |  | 0.16 |  | 0.78 | [58] |
| 1b |  | 450 |  |  | 34.3 | 49.6 | 13.9 | 3.03 | 0.37 |  | 3.6 |  | 37.6 |  | 134.1 | 0.36 |  | 0.24 |  | 0.81 |  |
| 1c |  | 500 |  |  | 60.3 | 79.9 | 9.77 | 3.50 | 0.20 |  | 8.2 |  | 48.9 |  | 399.5 | 0.84 |  | 0.27 |  | 3.32 |  |
| 1d |  | 550 |  |  | 97.6 | 148.3 | 16.9 | 4.33 | 0.33 |  | 8.8 |  | 51.2 |  | 449.4 | 0.94 |  | 0.35 |  | 5.67 |  |
| 1e |  | 600 |  |  | 55.3 | 39.3 | 0.67 | 0.23 | 0.03 |  | 58.7 |  | 22.3 |  | 1330 | 3.73 |  | 0.05 |  | 6.86 |  |
| 1f |  | 550+ST1 |  |  | 101.1 | 168.1 | 17.0 | 4.87 | 0.77 | 0.17 | 9.9 | 98.1 | 22.1 | 988.8 | 218.3 | 1.08 | 0.80 | 0.15 | 9.99 | 3.00 |  |
| 1g |  | 550+ST4 |  |  | 94.3 | 108.6 | 5.00 | 1.67 | 0.33 | 0.03 | 21.7 | 166.7 | 15.2 | 3620 | 329.1 | 1.96 | 0.86 | 0.07 | 24.65 | 3.36 |  |
| 1h |  | 550+ST8 |  |  | 79.3 | 60.3 | 1.93 | 0.70 | 0.07 | 0.01 | 31.2 | 193.0 | 27.6 | 6030 | 861.4 | 2.41 | 0.69 | 0.09 | 24.13 | 5.93 |  |
| 2a | Cellulose cuprammonium | na | 20 | 2 | 106.9 |  | 183.2 | 34.9 | 6.48 |  | 5.4 |  | 28.3 |  |  | 0.83 |  | 0.44 |  |  | [70] |
| 2b |  |  | 50 |  | 126.6 |  | 196.3 | 43.1 | 8.64 |  | 5.0 |  | 22.7 |  |  | 0.80 |  | 0.36 |  |  |  |
| 3a | Wood pulp (WP) | 550 | 30 | 2 |  | 940 | 190 | 54 | 4.1 | 4.6 | 13.2 | 41.3 | 46.3 | 204.3 | 229.3 | 2.20 | 0.85 | 0.73 | 9.77 | 10.04 | [57] |
| 3b | WP-CaO |  |  |  |  | 860 | 130 | 38 | 3.1 | 3.5 | 12.3 | 37.1 | 41.9 | 245.7 | 277.4 | 1.92 | 0.66 | 0.58 | 10.85 | 11.44 |  |
| 3c | WP-MgO |  |  |  |  | 1100 | 14 | 5.5 | 4.4 | 1.2 | 1.3 | 11.7 | 3.2 | 916.7 | 250.0 | 0.14 | 0.09 | 0.02 | 50.55 | 12.17 |  |
| 3d | WP-FeO |  |  |  |  | 280 | 110 | 30 | 8.3 | 4.0 | 3.6 | 27.5 | 13.3 | 70.0 | 33.7 | 0.54 | 0.46 | 0.17 | 1.12 | 0.65 |  |
| 3e | WP-SiO |  |  |  |  | 670 | 58 | 19 | 1.9 | 2.0 | 10.0 | 29.0 | 30.5 | 335.0 | 352.6 | 1.38 | 0.38 | 0.32 | 11.80 | 12.29 |  |
| 3f | WP-AgN |  |  |  |  | 1500 | 180 | 53 | 5.1 | 1.4 | 10.4 | 128.6 | 35.3 | 1071 | 294.1 | 1.72 | 2.58 | 0.54 | 78.15 | 17.64 |  |
| 3g | WP-CuN |  |  |  |  | 1100 | 81 | 25 | 2.3 | 0.7 | 10.9 | 115.7 | 35.2 | 1571 | 478.3 | 1.58 | 1.72 | 0.41 | 86.62 | 23.28 |  |
| 3h | WP-FeN |  |  |  |  | 1000 | 310 | 86 | 8.2 | 2.1 | 10.5 | 147.6 | 37.8 | 476.2 | 122.0 | 1.90 | 3.64 | 0.70 | 24.09 | 5.57 |  |
| 4a | WP-6day | 550+ST2 | 30 | 2 |  | 55000 | 28000 | 17000 | 29000 | 32000 | 0.6 | 0.9 | 1.0 | 1.7 | 1.9 | 0.28 | 0.12 | 0.09 | 3.21 | 1.29 | [89] |
| 4b | WP-14day |  |  |  |  | 940 | 190 | 54 | 4.2 | 4.6 | 12.9 | 41.3 | 45.2 | 204.3 | 223.8 | 2.15 | 0.85 | 0.71 | 9.77 | 9.80 |  |
| 4c | WP-74day |  |  |  |  | 860 | 150 | 48 | 5.7 | 1.3 | 8.4 | 115.4 | 26.3 | 661.5 | 150.9 | 1.37 | 2.16 | 0.38 | 29.20 | 6.22 |  |
| 4d | WP | 500 |  |  |  | 460 | 220 | 49 | 6.6 | 2.8 | 7.4 | 78.6 | 33.3 | 164.3 | 69.7 | 1.21 | 1.70 | 0.55 | 4.12 | 1.89 |  |
| 4e |  | 550 |  |  |  | 1100 | 310 | 90 | 8.6 | 2.8 | 10.5 | 110.7 | 36.0 | 393.9 | 127.9 | 1.91 | 2.73 | 0.67 | 21.72 | 6.22 |  |
| 4f |  | 550+ST2 |  |  |  | 940 | 190 | 54 | 4.2 | 4.6 | 12.9 | 41.3 | 45.2 | 204.3 | 223.8 | 2.15 | 0.85 | 0.71 | 9.77 | 9.80 |  |
| 4g |  | 650 |  |  |  | 1300 | 480 | 130 | 15 | 4.4 | 8.7 | 109.1 | 32.0 | 295.5 | 86.7 | 1.69 | 3.18 | 0.69 | 18.95 | 4.72 |  |
| 4h |  | 850 |  |  |  | 55 | 11 | 5.8 | 3.7 | 2.0 | 1.6 | 5.5 | 3.0 | 27.5 | 14.9 | 0.18 | 0.04 | 0.02 | 0.10 | 0.10 |  |
| 4i | WP-FeN 1.8 | 550+ST2 |  |  |  | 1000 | 310 | 86 | 8.2 | 2.1 | 10.5 | 147.6 | 37.8 | 476.2 | 122.0 | 1.90 | 3.64 | 0.70 | 24.09 | 5.57 |  |
| 4j | WP-FeN 4.1 |  |  |  |  | 330 | 30 | 14 | 6.0 | 5.5 | 2.3 | 5.5 | 5.0 | 60.0 | 55.0 | 0.30 | 0.06 | 0.04 | 1.12 | 1.19 |  |
| 5a | WP | 400 | 25 - 30 | 2 |  | 101 |  |  |  | <0.001 |  |  |  | >100000 |  |  |  |  | 98.64 |  | [59] |
| 5b |  | 550 |  |  |  | 1101 |  |  |  | 2.7 |  |  |  | 402 |  |  |  |  | 22.18 |  |  |
| 5c |  | 650 |  |  |  | 1388 |  |  |  | 1.2 |  |  |  | 1157 |  |  |  |  | 78.71 |  |  |
| 5d |  | 700 |  |  |  | 549 |  |  |  | <0.001 |  |  |  | >100000 |  |  |  |  | 2943 |  |  |
| 5e | WP-CuN 2 | 550 |  |  |  | 703 |  |  |  | 1.1 |  |  |  | 636 |  |  |  |  | 23.40 |  |  |
| 5f | WP-CuN 4 |  |  |  |  | 830 |  |  |  | 0.7 |  |  |  | 1227 |  |  |  |  | 52.46 |  |  |
| 6a | Cellulose acetate (CA) | 550+ST2 | 30 | 2 |  |  | 164 | 41 | 4.0 | 1.5 | 10.3 | 109.3 | 40 |  |  | 1.63 | 2.12 | 0.60 |  |  | [77] |
| 6b |  |  | 70 |  |  |  | 181 | 45 | 6.7 | 2.7 | 6.7 | 67.0 | 27 |  |  | 1.08 | 1.35 | 0.42 |  |  |  |

|  | Percursor | T_carbonization_ (ºC) | T_test_  (ºC) | P_feed_ (bar) | Permeability (barrer) | | | | | | | Ideal Selectivity | | | | | Robeson Index | | | | | Reference |
| --- | --- | --- | --- | --- | --- | --- | --- | --- | --- | --- | --- | --- | --- | --- | --- | --- | --- | --- | --- | --- | --- | --- |
|  |  |  |  |  | He | H_2_ | CO_2_ | O_2_ | N_2_ | CH_4_ | O_2_/N_2_ | | CO_2_/CH_4_ | CO_2_/N_2_ | H_2_/CH_4_ | H_2_/N_2_ | O_2_/N_2_ | CO_2_/CH_4_ | CO_2_/N_2_ | H_2_/CH_4_ | H_2_/N_2_ |  |
| 7a | CA-2h | 650+ST2 | 30 | 2 |  | 980 | 220 |  | 7 | 2.0 |  | | 110 | 31.4 | 490 | 140 |  | 2.38 | 0.52 | 24.34 | 6.30 | [76] |
| 7b | CA-8h |  |  |  |  | 250 | 29 |  | 0.8 | 0.25 |  |  | 116 | 36.3 | 1000 | 312.5 |  | 1.16 | 0.30 | 14.46 | 5.60 |  |
| 8a | CA | 550+ST2 | 30 | 2 |  | 500 | 150 | 37 | 3.8 | 1.6 | 9.7 | | 93.8 | 39.5 | 312.5 | 131.6 | 1.51 | 1.76 | 0.57 | 8.45 | 3.76 | [90] |
| 8b |  |  |  |  |  | 650 | 280 | 68 | 10 | 4.0 | 6.8 | | 70.0 | 28.0 | 162.5 | 65.0 | 1.18 | 1.66 | 0.50 | 5.57 | 2.22 |  |
| 9a | CA | 550+ST2 | 20 | 5 |  |  |  | 3.0 | 0.17 |  | 17.5 | |  |  |  |  | 1.75 |  |  |  |  | [96] |
| 9b |  |  | 25 |  |  |  |  | 3.1 | 0.09 |  | 34.4 | |  |  |  |  | 3.46 |  |  |  |  |  |
| 9c |  |  | 35 |  |  |  |  | 4.5 | 0.24 |  | 18.8 | |  |  |  |  | 2.02 |  |  |  |  |  |
| 9d |  |  | 45 |  |  |  |  | 5.8 | 0.29 |  | 19.8 | |  |  |  |  | 2.22 |  |  |  |  |  |
| 9e |  |  | 50 |  |  |  |  | 6.4 | 0.33 |  | 29.2 | |  |  |  |  | 3.33 |  |  |  |  |  |
| 9f |  |  | 68 |  |  |  |  | 9.9 | 0.54 |  | 18.3 | |  |  |  |  | 2.26 |  |  |  |  |  |
| 10a | CA | 550+ST2 | 23 | 5 |  |  | 0.006 |  | 0.003 |  |  | | 6 | 2 |  |  |  | 0.00 | 0.00 |  |  | [7] |
| 10b |  |  |  |  |  |  | 1.44 |  | 0.029 |  |  |  | 147 | 49 |  |  |  | 0.47 | 0.14 |  |  |  |
| 10c |  |  |  |  |  |  | 67.6 |  | 1.83 |  |  |  | 111 | 37 |  |  |  | 1.54 | 0.41 |  |  |  |
| 10d |  |  |  |  |  |  | 75.6 |  | 1.76 |  |  |  | 129 | 43 |  |  |  | 1.86 | 0.49 |  |  |  |
| 10e | CA-CVD |  |  |  |  |  | 10.8 |  | 0.28 |  |  |  | 114 | 38 |  |  |  | 0.79 | 0.22 |  |  |  |
| 10f |  |  |  |  |  |  | 318 |  | 3.88 |  |  |  | 246 | 82 |  |  |  | 6.13 | 1.54 |  |  |  |
| 10g |  |  |  |  |  |  | 655 |  | 40.9 |  |  |  | 48 | 16 |  |  |  | 1.57 | 0.38 |  |  |  |
| 10h |  |  |  |  |  |  | 876 |  | 79.6 |  |  |  | 33 | 11 |  |  |  | 1.21 | 0.29 |  |  |  |
| 11a | CA-65NMP | 650+ST2 | 23 | 5 |  |  | 260 |  | 4.9 |  |  | | 160 | 53.3 |  |  |  | 3.69 | 0.93 |  |  | [80] |
| 11b | CA-85NMP |  |  |  |  |  | 10 |  | 0.23 |  |  |  | 130 | 43.3 |  |  |  | 0.87 | 0.24 |  |  |  |
| 11c | CA-85DMSO |  |  |  |  |  | 4 |  | 4 |  |  |  | 3 | 1 |  |  |  | 0.01 | 0.00 |  |  |  |
| 11d | CA-95DMSO |  |  |  |  |  | 19 |  | 0.26 |  |  |  | 220 | 73.3 |  |  |  | 1.88 | 0.52 |  |  |  |
| 11e | CA-80RH |  |  |  |  |  | 59 |  | 0.35 |  |  |  | 500 | 166.7 |  |  |  | 6.57 | 1.74 |  |  |  |
| 11f | CA-65RH |  |  |  |  |  | 300 |  | 5.6 |  |  |  | 160 | 53.3 |  |  |  | 3.90 | 0.98 |  |  |  |
| 11g | CA-55RH |  |  |  |  |  | 420 |  | 10.5 |  |  |  | 120 | 40 |  |  |  | 3.32 | 0.83 |  |  |  |
| 12a | CA-N2 | 650+ST2 | 20 - 23 | 5 |  |  | 410 |  | 17.3 |  |  | | 71 | 23.7 |  |  |  | 1.95 | 0.48 |  |  | [81] |
| 12b |  |  |  |  |  |  | 210 |  | 3.9 |  |  |  | 160 | 53.3 |  |  |  | 3.40 | 0.86 |  |  |  |
| 12c |  |  |  |  |  |  | 32 |  | 0.38 |  |  |  | 250 | 83.3 |  |  |  | 2.61 | 0.70 |  |  |  |
| 12d | CA-CO2 |  |  |  |  |  | 1.7 |  | 0.004 |  |  |  | 1300 | 433.3 |  |  |  | 4.45 | 1.33 |  |  |  |
| 12e |  |  |  |  |  |  | 75 |  | 1.6 |  |  |  | 140 | 46.7 |  |  |  | 2.02 | 0.53 |  |  |  |
| 12f |  |  |  |  |  |  | 80 |  | 2.4 |  |  |  | 100 | 33.3 |  |  |  | 1.48 | 0.39 |  |  |  |
| 13a | CP | 400 | 25 | 1 | 5.43 | 8.35 | 3.39 | 0.73 | 0.07 | 0.008 | 10.4 | | 423.8 | 48.4 | 1043.8 | 119.3 | 0.81 | 1.88 | 0.19 | 0.70 | 0.22 | [75] |
| 13b |  | 500 |  |  | 10.24 | 18.94 | 8.21 | 0.95 | 0.06 | nd | 15.8 | | nd | 136.8 | nd | 315.7 | 1.29 | 9.66 | 0.72 | 7.48 | 1.00 |  |
| 13c |  | 550 |  |  | 17.26 | 32.59 | 13.03 | 1.33 | 0.07 | 0.01 | 19.0 | | 1303.0 | 186.1 | 3259.0 | 465.6 | 1.64 | 100.1 | 1.15 | 45.00 | 2.12 |  |
| 12d |  | 600 |  |  | 11.78 | 24.90 | 2.57 | 0.78 | <0.001 | <0.001 | >800 | | >2600 | >2600 | >25000 | >25000 | 63.02 | 1.88 | 9.18 | 0.70 | 94.76 |  |
| 14a | WP+IL | 550 | 25 | 1 | 126 | 206 | 13.4 | 5.15 | 0.16 |  | 32.3 | |  | 83.8 |  | 1287.5 | 3.55 |  | 0.52 |  | 20.26 | [30] |
| 14b |  | 600 |  |  | 121 | 174 | 4.18 | 2.19 | 0.09 |  | 24.3 | |  | 46.4 |  | 1933.3 | 2.30 |  | 0.19 |  | 27.16 |  |
| 15a | MCC+IL | 600+ST2 | 25 | 2 |  |  | 239 | 74 | 5.69 | 1.28 | 13 | | 186 | 4.45 |  |  | 2.29 | 4.16 | 0.08 |  |  | [73] |
| 15b |  |  |  |  |  |  | 264 | 68 | 6.73 | 1.53 | 10.1 | | 173 | 4.40 |  |  | 1.75 | 4.01 | 0.08 |  |  |  |
| 15c |  |  |  |  |  |  | 86 | 27 | 1.58 | 0.27 | 17 | | 321 | 5.85 |  |  | 2.50 | 4.87 | 0.07 |  |  |  |

CP – cellophane paper; WP – wood pulp; CA – cellulose acetate; ST – soak time (hours); na – not available; nd – not determined; CVD – chemical vapor deposition; RH – relative humidity; MCC – microcrystalline cellulose
